# Electrical spin pumping in exchange-coupled molecules

**DOI:** 10.1126/sciadv.aei0428

**Published:** 2026-11-13

**Authors:** Paul Greule, Wantong Huang, Kwan Ho Au-Yeung, Máté Stark, Johannes Schwenk, Christoph Sürgers, Wolfgang Wernsdorfer, Philip Willke

**Affiliations:** ^1^ Physikalisches Institut (PHI), Karlsruhe Institute of Technology (KIT), Karlsruhe, Germany.; ^2^ Center for Integrated Quantum Science and Technology (IQST), Karlsruhe Institute of Technology, Karlsruhe, Germany.; ^3^ Institute for Quantum Materials and Technologies (IQMT), Karlsruhe, Germany.

## Abstract

Electron spins in single molecules are a promising platform for quantum information processing. However, their practical implementation as qubits requires reliable control at the single-entity level, including an efficient state initialization. Here, we demonstrate the remote, all-electrical spin pumping of the electron spin in single molecules: Using electron spin resonance scanning tunneling microscopy, we investigate coupled pairs of S = 1/2 molecules, where one molecule serves as a readout and pumping unit for the neighboring one. We show that the exchange interaction between them enables angular momentum transfer, which allows for the control of the remote spin state via the direction and magnitude of the spin-polarized tunneling current and the exchange coupling strength. These results establish a general, all-electrical approach for remote spin pumping that is transferable to a wider range of spin-based quantum architectures.

## INTRODUCTION

The controlled initialization of quantum states is a central requirement for quantum technologies (*1*), ranging from quantum computing to nanoscale sensing and simulation. At thermal equilibrium, however, the occupation of quantum states follows the Boltzmann distribution and is therefore fixed by the ratio between energy splitting and temperature, which fundamentally limits the achievable initialization fidelity and requires the use of low temperatures in the millikelvin regime.

In this regard, a variety of approaches to actively control the occupation of quantum states has been developed across quantum platforms. Examples are energy-selective tunneling into an electron reservoir for quantum dots (*2*) as well as optical pumping for solid-state color centers (*3*) and trapped ions (*4*). Optical pumping has also been proposed and is being explored as a strategy for initializing molecular spin qubits (*5*–*7*). The latter have attracted growing interest as quantum building blocks due to their chemical tunability, intrinsic multilevel structure, and potential to scale up through self- assembly (*8*–*11*). Yet, their controlled initialization, particularly at the level of individual molecules, remains a major challenge. Addressing this requires approaches that are efficient, fast, and compatible with device integration. In this context, electrical control is appealing, as it provides direct addressability and high-speed operation without the need for extended optical setups.

To access and manipulate individual spins with atomic precision, scanning tunneling microscopy combined with electron spin resonance (ESR-STM) has emerged as a powerful platform (*12*). By combining atomic-scale spatial resolution alongside energy resolution in the megahertz regime, ESR-STM enables the detection and coherent control of spin transitions in individual atoms, molecules, and solid-state defects (*12*–*18*). Recent works have further demonstrated the controlled hyperpolarization of the nuclear spin of single Cu and Ti atoms via the spin-polarized tunneling current and transfer of angular momentum (*19*–*21*), which is mediated by the hyperfine interaction. This approach offers a promising route for the control of electron spins in molecules.

In this work, we demonstrate current-induced polarization of a remote electron spin in a magnetic molecule. Using pairs of iron-phthalocyanine molecules coupled to additional iron atoms, referred to as Fe-FePc complexes, we realize a system in which one molecule is addressed by the STM tip and serves as a sensing and pumping unit to remotely change and probe the state occupation of a neighboring molecule. By controlling the magnitude and polarity of the spin-polarized tunneling current, we drive the remote spin into a non-thermal steady state, including occupation inversion by transferring angular momentum from the magnetic STM tip. Last, we demonstrate how this electrical pumping can be varied by tuning the exchange interaction between spins and discuss its broader implications for spin-qubit platforms.

## RESULTS

### Investigated spin system

The experimental platform consists of iron phthalocyanine (FePc) molecules and single iron (Fe) atoms adsorbed on two monolayers of magnesium oxide (MgO) on a Ag(100) crystal substrate (see Fig. 1A). The thin MgO film serves as a decoupling layer to protect the single spin hosts, i.e., the magnetic molecules and atoms, from substrate electrons while maintaining sufficient conductivity for a finite tunneling current to probe the surface spins (*22*). Using tip-assisted atom manipulation, single Fe atoms are combined with FePc molecules to form Fe-FePc complexes, where the additional Fe atom is positioned below one of the benzene rings of the FePc molecule (*23*). This coupled spin system exhibits an enhanced spin lifetime compared to pristine FePc molecules as shown in (*23*). It moreover showed coherent spin dynamics, which was demonstrated by Rabi and Ramsey measurements in previous works (*23*, *24*) and is therefore a suitable spin system to investigate. As it forms an effective S = 1/2 system, we treat it as such in the following to simplify the discussion. Using these units as building blocks, molecule pairs are assembled by positioning two Fe-FePc complexes in close proximity with the STM tip as shown in Fig. 1B (also see fig. S1).

**Fig. 1.**
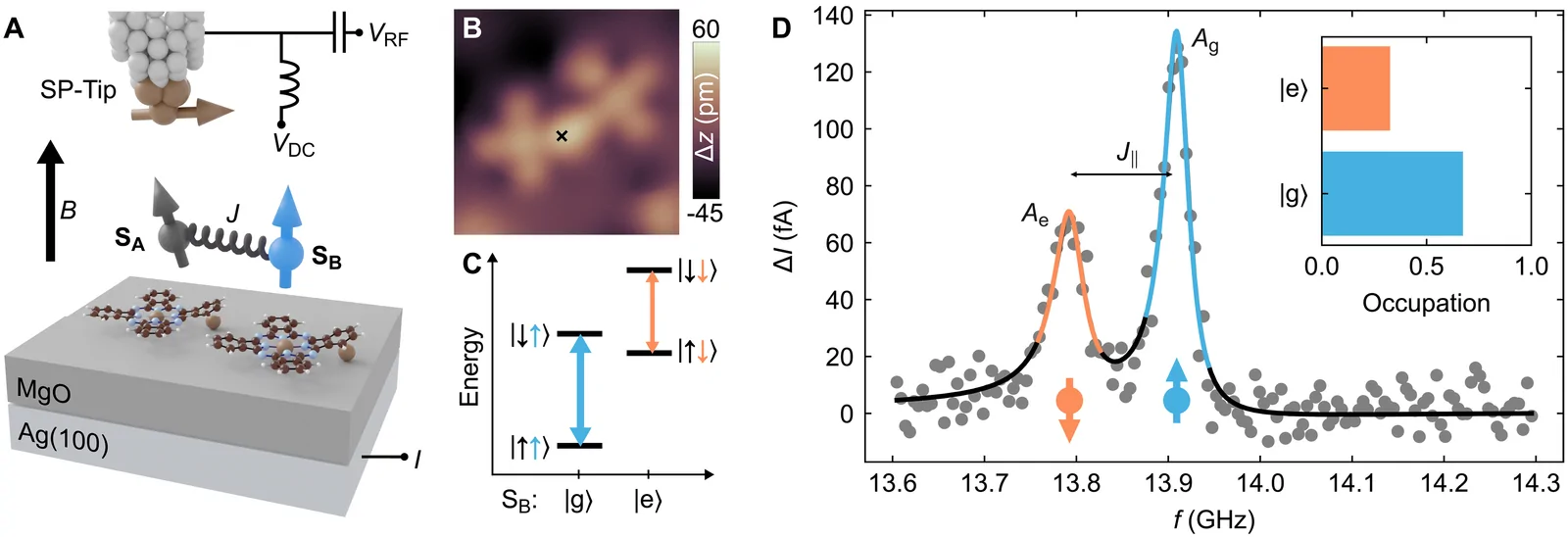
Measurement scheme. (**A**) Schematic illustration of the tunnel junction with two molecule spins assembled on MgO/Ag(100). For ESR measurements, an external magnetic field B is applied along the *z* axis and a magnetic tip apex is used. A DC bias voltage $V_{\text{DC}}$ and a radio-frequency voltage $V_{\text{RF}}$ are applied to the tip, which is positioned above the sensing spin S_A_ to probe and drive its spin state. S_A_ is exchange coupled to a second, remote spin S_B_. (**B**) STM topography of a molecule pair (image conditions: $3\;\text{nm}\times 3\;\text{nm},\;I=10\;\text{pA},V_{\text{DC}}=-100\;\text{mV}$). The cross marks the measurement position used in (D) (the topography is also shown in Fig. 4A and fig. S1). (**C**) Energy-level diagram of the spin system, showing the configurations with the remote spin S_B_ in the ground state ∣g〉 (left) and the excited state ∣e〉 (right). The two ESR transitions are indicated by colored arrows of different thickness. (**D**) ESR frequency sweep of the molecule pair in (B), showing two resonances associated with the two transitions shown in (C) corresponding to the two spin orientations of S_B_. The peak amplitudes $A_{g}$ and $A_{e}$ are obtained from fitting a double Fano function as indicated. Inset: Extracted state occupation of S_B_ $\left(p_{g}=67\%,p_{e}=33\%\right)$ (ESR conditions: $G_{\text{set}}=0.13\;\text{nS},V_{\text{DC}}=-50\;\text{mV},\;B=458\;\text{mT},\;V_{\text{RF}}=10\;\text{mV}$).

In the following experiments, the cryostat was operated at a base temperature of approximately 50 mK. An external magnetic field B is applied perpendicular to the sample surface (along the *z* axis), which defines the quantization axis of the molecule spins. The magnetic STM tip, which is created by transferring several Fe atoms to the tip apex, is positioned above one Fe-FePc complex (sensing spin S_A_). This spin then senses the neighboring Fe-FePc complex (remote spin S_B_) via the exchange interaction between them. For that, we probe both spins in ESR measurements, where an AC voltage $V_{\text{RF}}$ (in the radio-frequency range) is applied to the STM tip. The spin readout is established by applying a DC bias voltage $V_{\text{DC}}$ and measuring the spin-polarized tunneling current between magnetic tip and sample (see Fig. 1A).

### State occupation measurement

To describe the coupled spin system of the two Fe-FePc complexes in greater detail, we use an effective spin Hamiltonian (*15*, *23*, *25*, *26*) (see section S1)

$$H=-g\mu_{B}\left(B+B_{\text{tip}}\right)S_{A,z}-g\mu_{B}{\mathrm{BS}}_{B,z}+J_{⊥}\left(S_{A,x}S_{B,x}+S_{A,y}S_{B,y}\right)+J_{∥}S_{A,z}S_{B,z}$$ (1)

The first two terms describe the Zeeman energies of S_A_ and S_B_ with the *g* factor of the Fe-FePc complexes, g=2.01±0.05 (*23*). The local magnetic field $B_{\text{tip}}$ originating from the magnetic tip allows to detune the two spins so that the eigenstates of the system are well described by the Zeeman product states of the two individual spins. The detuning is given by $\delta =g\mu_{B}B_{\text{tip}}$. The last two terms account for the interaction between S_A_ and S_B_, which is modeled here by an anisotropic exchange coupling with parallel and perpendicular component $J_{∥}$ and $J_{⊥}$ to the quantization axis, respectively. For the proposed spin-pumping mechanism, the system must be operated in the weak coupling regime where $|J_{⊥}|<\delta$. We note that the weaker dipole-dipole interaction (≈50 MHz) is not separately considered for simplicity and is absorbed in the effective exchange parameters.

The resulting energy-level diagram of the coupled spin system is shown in Fig. 1C: Two ESR transitions are possible for S_A_ that depend on the state of S_B_ (*27*). Figure 1D shows a corresponding frequency sweep. The spectrum reveals two pronounced resonance frequencies separated by $\Delta f=|J_{∥}|$ (where we set Planck’s constant h=1, see section S1 for details). According to Fig. 1C, the higher (lower) peak corresponds to the remote spin S_B_ occupying the ground state ∣g〉 (excited state ∣e〉). Moreover, the amplitudes of the two resonances $A_{g}$ and $A_{e}$ are directly proportional to the state occupations $p_{g},p_{e}$ of S_B_ following the relation $A_{g}/A_{e}=p_{g}/p_{e}$ (with $p_{g}+p_{e}=1$) (*27*). Therefore, the ESR spectrum provides a direct measure of the state occupation of the remote spin.

### Electrically controlled spin polarization

Figure 2 presents a series of ESR frequency sweeps recorded at different $V_{\text{DC}}$ at fixed tip height. Thus, the tunneling current $I=G_{\text{set}}V_{\text{DC}}$ increases where $G_{\text{set}}$ is the constant conductance setpoint of the measurement. For negative voltages, corresponding to electron tunneling from the sample to the tip, the relative amplitudes of the two ESR transitions change markedly: With decreasing $V_{\text{DC}}$, the peak amplitudes become progressively more equal in height, eventually leading to a regime where the transition associated with S_B_ being in the excited state ∣e〉 becomes slightly more dominant. In contrast, applying a positive bias voltage (current from tip to sample) has only a minor influence on the relative peak amplitudes. In Fig. 3C, we demonstrate this effect again by plotting obtained occupations $p_{g}$ and $p_{e}$ as a function of $V_{\text{DC}}$ and I (different dataset than Fig. 2). Here, the occupations of the two states gradually approach each other for negative $V_{\text{DC}}$ and eventually invert at ≈−60 mV. In contrast, for positive $V_{\text{DC}}$, the state occupations show only a weak dependence.

**Fig. 2.**
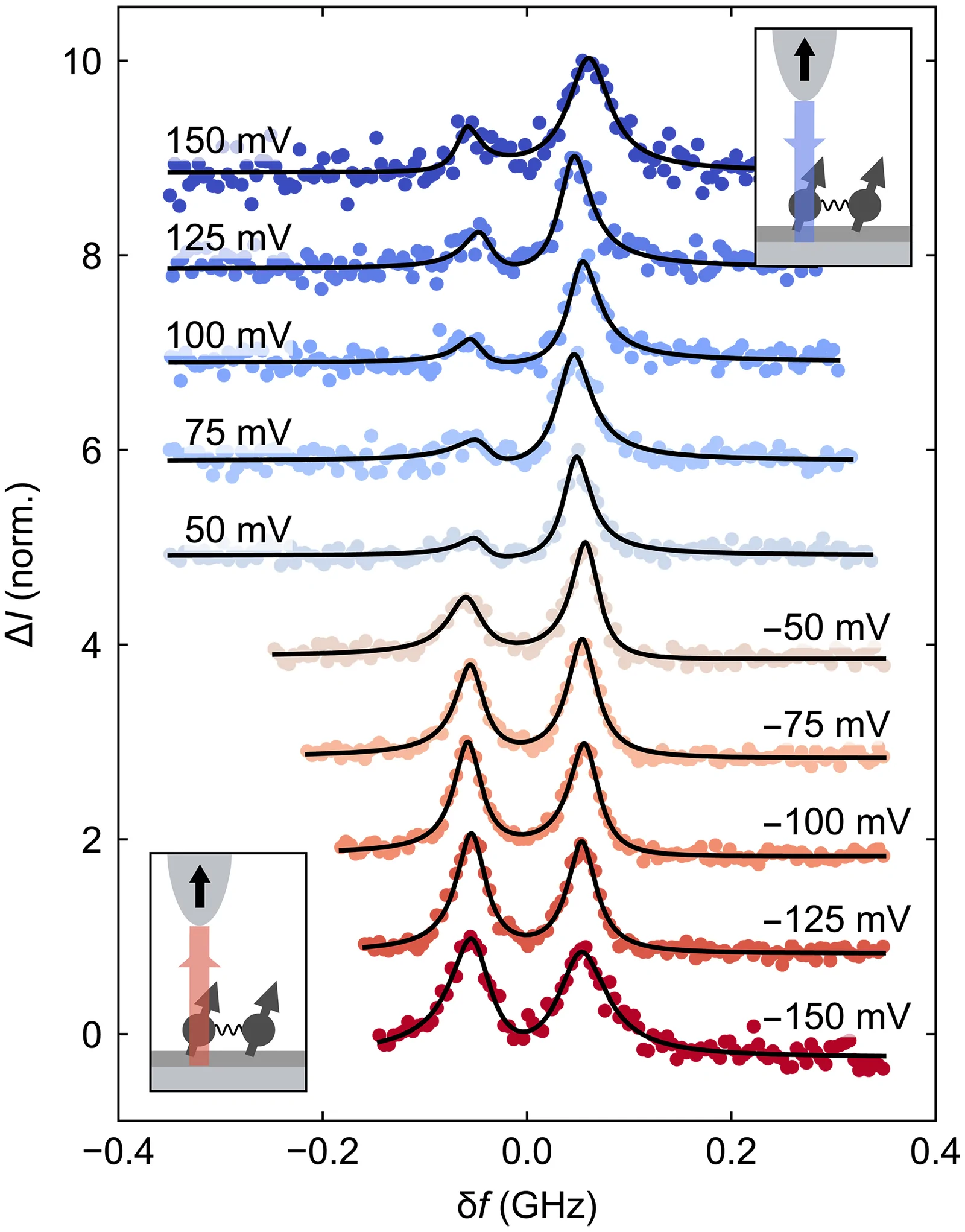
Voltage dependence of the ESR spectra. Change in tunneling current ΔI as a function of radio-frequency shift $\delta f=f-f_{0}$ for different $V_{\text{DC}}$ at constant conductance setpoint $G_{\text{set}}$ (ESR conditions: same as in Fig. 1D). The traces are normalized and vertically offset for clarity. In addition, they are centered at their resonance frequencies to compensate for the voltage induced frequency shift arising from spin-electric coupling (*24*). Colored dots show the experimental data and black solid lines represent fits to a double Fano function. The insets illustrate the tunnel junction with the direction of the tunneling current for positive and negative $V_{\text{DC}}$.

**Fig. 3.**
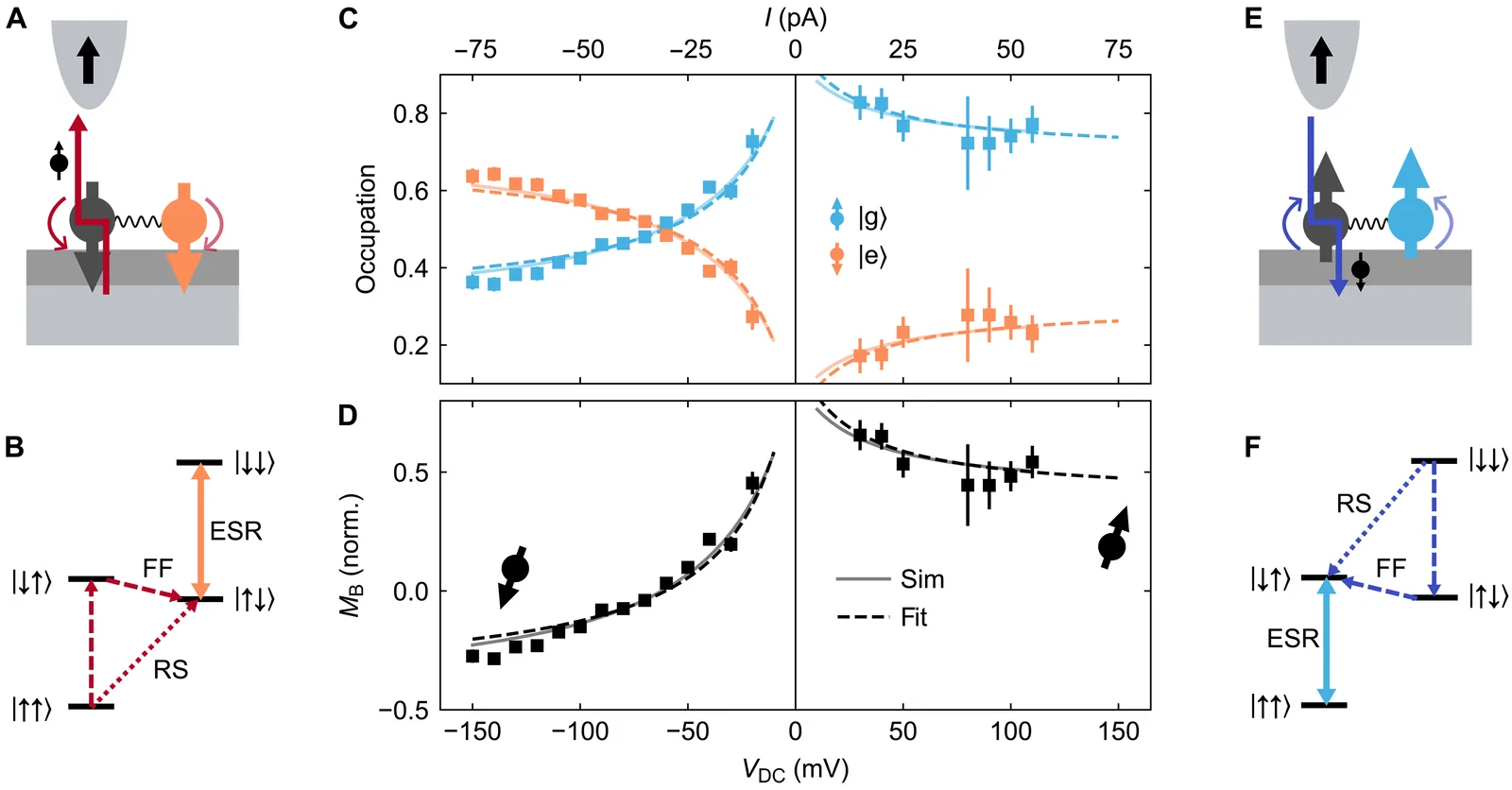
Remote spin pumping in a molecule pair. (**A**) Spin excitation: Schematic of the tunnel junction illustrating the remote spin excitation toward ∣↓↓〉 of the exchange-coupled spins driven by the spin-polarized tunneling current for I<0. (**B**) Energy-level diagram of the coupled spin system. The flip-flop (FF) and remote scattering (RS) transitions that dominate for $V_{\text{DC}}<0$ and drive the system into the nonequilibrium steady state are indicated by red arrows. Reverse transitions are omitted for clarity. ESR detection is shown in orange. (**C**) Extracted occupations of the ground ∣g〉 and excited state ∣e〉 of S_B_ as a function of $V_{\text{DC}}$ and I at constant $G_{\text{set}}$. Data points are obtained from ESR frequency sweeps as presented in Fig. 1D (ESR conditions: $G_{\text{set}}=0.5\;\text{nS},B=450\;\text{mT},V_{\text{RF}}=12\;\text{mV}$). (**D**) Corresponding magnetization $M_{B}$ plotted over $V_{\text{DC}}$ and I. Solid lines in (C) and (D) represent fits to the full rate simulation (simulation parameters: $T=0.21\pm 0.12\;K,G_{\text{st}}/G_{\text{ss}}=0.17\pm 0.03,\;\eta =0.34\pm 0.04$). Dashed lines represent fits to the analytical formula given by Eq. 4: $I_{0}=-30.2\pm 1.8\;\text{pA},\;\eta =0.39\pm 0.02$. For negative (positive) values ∣e〉 (∣g〉) is primarily occupied as illustrated by the inserted sketches. (**E**) Spin relaxation: Schematic of the tunnel junction for I>0 illustrating the remote spin relaxation into ∣↑↑〉. (**F**) Analogous to (B), energy-level diagram with the FF and RS transitions that dominate for $V_{\text{DC}}>0$ shown in blue and ESR detection in light blue.

From the state occupation of the remote spin, we derive its magnetization as $M_{B}=p_{g}-p_{e}$ (Fig. 3D): $M_{B}=1\;\left(M_{B}=-1\right)$ corresponds to a full occupation of the ground (excited) state, and thus, $M_{B}$ intuitively reflects the spin state polarization.

The observed occupation inversion for negative voltages cannot be reconciled with a thermal Boltzmann distribution of the spin system’s state occupations as reported for other systems (*27*, *28*). Further, the strong asymmetry with respect to the tunnel direction highlights the nonequilibrium nature of the remote spin state occupation and indicates a bias-polarity–dependent control mechanism. Moreover, by performing setpoint-dependent measurements, we find that the spin pumping depends on the induced tunneling current I instead of the explicit bias voltage $V_{\text{DC}}$ (see fig. S7).

### Inelastic tunneling theory

To explain the observed change in state occupation, we use a model of tunneling current induced spin pumping [see (*29*)]. This has been successfully applied to single atom nuclear spins (*19*, *20*): In these experiments, the spin imbalance of the spin-polarized tip allows to polarize the electron spin via the tunneling current while the hyperfine interaction couples the electron and nuclear spin. The latter subsequently enables spin transfer between the two. Nevertheless, despite a large number of coupled spin systems reported in literature (*13*, *15*, *25*, *26*), this has not been observed for electron or molecular spin systems.

To describe the influence of the tunneling current on the spin state occupation, we consider second-order tunneling processes between the spin system and the electrodes, following the framework introduced in (*30*–*33*). In this approach, the surface spins are treated as an open quantum system that is weakly coupled to the electronic reservoirs formed by the STM tip and the substrate (*34*, *35*). Inelastic tunneling events induce transitions between the spin eigenstates and thereby redistribute the state occupations. In the tunnel junction geometry used in this work, the sensing spin S_A_ is subject to inelastic scattering from both the tip and the sample, whereas the remote spin S_B_ is only subject to electrons originating from the sample electrode. The magnetic tip electrode is modeled as a spin-polarized electron reservoir characterized by the polarization parameter η, which quantifies the imbalance between spin up (down) electron densities $n_{↑}^{t}=\frac{1+\eta }{2}\;\left(n_{↓}^{t}=\frac{1-\eta }{2}\right)$. In contrast, the sample electrode has equal electron densities $n_{↑}^{s}=n_{↓}^{s}=\frac{1}{2}$. On the basis of these tunneling processes and their rates, the state occupation of the spin system can be obtained numerically from the steady-state solution of the corresponding rate equation (see section S2).

To additionally establish an intuitive picture, we derive an analytical solution in the following: At sufficiently large bias voltages, $|{\mathrm{eV}}_{\text{DC}}|>E_{i}>k_{B}T$, with $E_{i}$ being the eigenenergies of the spin system, inelastic scattering with tunneling electrons dominates over thermally activated transitions. This drives the spin system out of thermal equilibrium and enables current induced spin pumping. Here, the spin polarization of the tip causes an asymmetry between relaxation and excitation rates. For a given tip polarization η, the direction of I determines whether relaxation or excitation is enhanced. This is illustrated in Fig. 3, A and E, where for I<0 (I>0) the surface spin is mainly excited (relaxed) by the tunneling electrons. This process competes with scattering processes in which electrons originate and return to the same electrode, namely tip-tip and sample-sample scattering: They drive the system toward thermal equilibrium. However, for S_A_, these processes are negligible due to the dominant tunneling current, whereas for S_B_, relaxation via substrate electrons (sample-sample scattering) remains a dominant process.

Although the tunneling electrons couple directly only to the sensing spin S_A_, a finite state mixing between S_A_ and S_B_ enables the spin transfer to the remote spin. For that, we identify two contributing tunneling processes (Fig. 3, B and F): (i) excitation of S_A_ is followed by a flip-flop (FF) interaction with the remote spin. We note that the FF transition itself is symmetric. Its contribution to excitation or relaxation of S_B_ becomes directional through the current-dependent occupation of S_A_; (ii) a tunneling electron directly induces a transition of the remote spin via the small but finite state mixing (referred to as remote scattering RS). We can quantify the relative probabilities of these processes by computing the transition matrix elements of the corresponding spin states

$$M_{\text{FF}}=\frac{J_{⊥}^{2}}{4\left(\delta^{2}+J_{⊥}^{2}\right)}$$ (2)

$$M_{\text{RS}}=\frac{1}{2}-\frac{\delta }{2\sqrt{\delta^{2}+J_{⊥}^{2}}}$$ (3)

They depend on $J_{⊥}$ and the detuning δ, i.e., the difference in Zeeman energy of S_A_ and S_B_ resulting from the magnetic tip field $B_{\text{tip}}$ (*15*). Both parameters can be obtained experimentally as shown in fig. S3.

On the basis of the dominant processes described above, we derive an analytical expression for the remote spin magnetization $M_{B}$ as a function of the tunneling current I (see section S3 for details)

$$M_{B}\left(I\right)=\eta \frac{I-I_{0}}{|I|+|\eta I_{0}|}\;\text{with}\;I_{0}=-\frac{G_{\mathrm{ss}}g\mu_{B}B}{2eM\eta }$$ (4)

where $I_{0}$ is the characteristic tunneling current at which $M_{B}\left(I_{0}\right)=0$, i.e., corresponding to equal state occupations $p_{e}=p_{g}.$ $I_{0}$ thus provides a measure of the spin pumping efficiency. This process is facilitated by large FF and remote scattering probabilities $M=M_{\text{FF}}+M_{\text{RS}}$ and large spin polarization of the tip 𝜂. In contrast, spin relaxation via the surface, which is proportional to the conductance $G_{\text{ss}}$ and the Zeeman energy $g\mu_{B}B$, hinders the spin pumping. In the limit of large tunneling currents $\left(|I|\gg |I_{0}|\right),M_{B}$ approaches a steady state determined solely by the tip polarization $\eta \;\left(M_{B}\to -\eta \;\text{for}\;V_{\text{DC}}<0\;\text{and}\;M_{B}\to \eta \;\text{for}\;V_{\text{DC}}>0\right)$. Consequently, the achievable degree of magnetization is limited by the spin polarization of the tip, which was found to lie in the range 0.2≤∣η∣≤0.5 for the measurements presented here. This variation is expected, as the spin polarization of the tip depends on the microscopic structure of the individual tip apex (*32*, *36*).

In Fig. 3 (C and D), we plot the analytical expression (Eq. 4) and the numerical solution of the rate equation model along with the experimental data that show excellent agreement. For the discussed dataset, we find a positive tip polarization η>0 that favors excitations when tunneling from sample to tip, while also the opposite behavior can be obtained for magnetic tips with η<0 (see fig. S6). Further, the numerical solution of the full rate equation allows us to estimate a spin temperature of T≈0.2 K for the system in the absence of any tunneling current ($V_{\text{DC}}=0$), where the system is in thermal equilibrium.

### Tuning the exchange interaction

The model we established in Eq. 4 illustrates that $M_{B}$ depends on the transition matrix elements for inelastic scattering processes between S_A_ and S_B_, $M_{\text{RS}}$ and $M_{\text{FF}}$. As both depend on the degree of state mixing, we expect a direct dependence on the perpendicular exchange interaction $J_{⊥}$ (Eqs. 2 and 3). Therefore, we construct pairs of Fe-FePc complexes with different spacing and orientation by tip-assisted atom manipulation (Fig. 4A), which results in different exchange couplings. For FePc pairs, it was shown that the exchange interaction is mediated by the ligands of the molecules (*15*). Because the Fe-FePc complexes studied here share the same molecular framework, we assign the observed coupling to a similar ligand-mediated exchange mechanism.

**Fig. 4.**
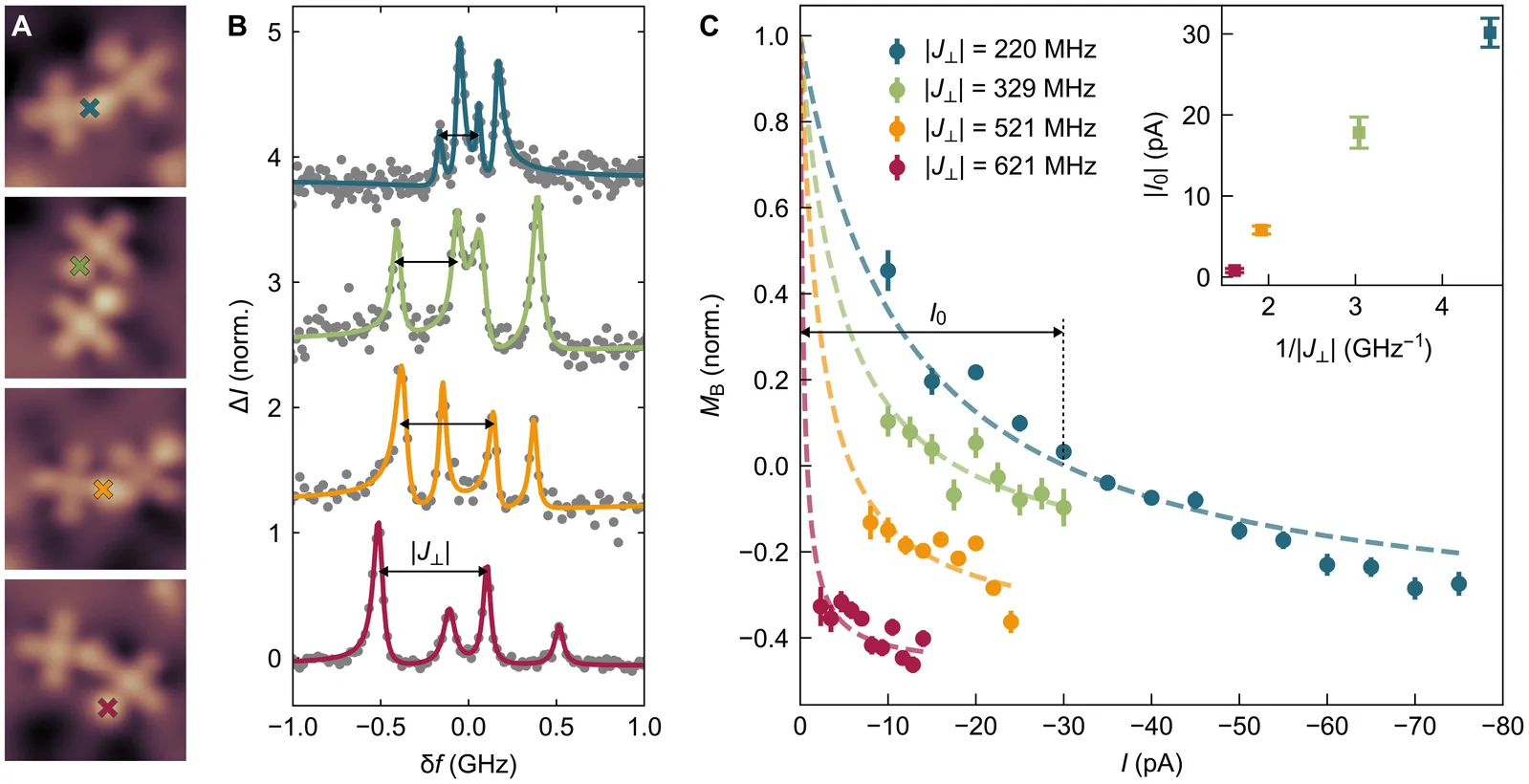
Spin pumping for different exchange interaction strengths. (**A**) STM topographies of four molecule pairs. Colored crosses mark the measurement positions used in (B) and (C) (image conditions: $3\;\text{nm}\times 3\;\text{nm},I=10\;\text{to}\;20\;\text{pA},V_{\text{DC}}=\pm 100\;\text{mV}$; the topography shown at the top is the same as in Fig. 1B). (**B**) Corresponding ESR spectra recorded at the avoided level crossing (δ=0) showing ΔI as a function of radio-frequency shift δf. Gray dots represent experimental data, and solid lines are fits using a four peak Fano function. For clarity, the spectra are vertically offset. Four resonance peaks are resolved from which the perpendicular exchange interactions $|J_{⊥}|=f_{\text{III}}-f_{I}=f_{\text{IV}}-f_{\text{II}}$ are extracted as indicated for each trace (also see section S1 and table S1). Note that the transition labels $f_{I}\;\text{to}\;f_{\text{IV}}$ are not generally ordered by increasing frequency, see section S1 (ESR conditions: $G_{\text{set}}=0.30\;\text{to}\;0.68\;\text{nS},|V_{\text{DC}}|=40\;\text{to}\;60\;\text{mV},B=473\;\text{to}\;495\;\text{mT},V_{\text{RF}}=10\;\text{to}\;12\;\text{mV}$). (**C**) Remote spin magnetization $M_{B}$ as a function of the induced tunneling current for the four pairs. Dashed lines are fits to the experimental data (circles) based on Eq. 4. We note that each trace was obtained with a different tip (ESR conditions: $G_{\text{set}}=0.3\;\text{to}\;0.5\;\text{nS},B=450\;\text{to}\;495\;\text{mT},V_{\text{RF}}=12\;\text{mV}$). Stronger exchange coupling leads to an enhanced spin pumping efficiency, reflected by a reduced characteristic current $I_{0}$, which is obtained from the fits (indicated for the blue trace). The inset displays $|I_{0}|$ plotted over $1/|J_{⊥}|$ showing a monotonic increase.

To determine $|J_{⊥}|$ experimentally, we perform setpoint-dependent ESR frequency sweeps. Here, the tip is moved closer to the surface to tune the tip field until the Zeeman energies of the two spins become equal (δ=0). The corresponding ESR spectra for the four molecule pairs are shown in Fig. 4B. Each spectrum exhibits four resonance peaks instead of the two resonances found for δ>0 (Figs. 1 to 3). This arises because, for δ=0, the eigenstates become mixed states that allow Δm=1 transitions between all eigenstates. $|J_{⊥}|$ can then be extracted from the transition frequencies given by the resonance positions (see section S1 and figs. S2 and S3 for details). The determined coupling strengths, on the order of several hundred megahertz, are comparable to those reported for similar spin systems, including pristine FePc molecules (*15*), Ti atoms (*25*), and molecule-atom heterodimers (*15*, *37*).

Figure 4C shows the magnetization $M_{B}\left(I\right)$ for all four pairs of complexes in the detuned regime δ>0 analogous to Fig. 3D. We find that pairs with stronger exchange coupling exhibit a substantially enhanced spin pumping efficiency, characterized by a faster approach to saturation with increasing I. This trend is shown in the inset of Fig. 4C where $I_{0}$ is plotted as a function of $1/|J_{⊥}|$. For the given parameter range, the monotonic increase is in good agreement with Eqs. 2 to 4, where a larger $|J_{⊥}|$ increases the degree of state mixing and thereby enhances the remote excitation and FF process. As a consequence, engineering and tuning the exchange coupling, within the range $|J_{⊥}|<\delta <g\mu_{B}B$, provides an additional strategy for controlling the spin pumping, complementary to the voltage controlled tunneling current.

From the relation between $I_{0}$ and $M\left(J_{⊥}\right)$, we find an estimate for the remote spin’s lifetime $T_{1}\approx 1\;\text{to}\;10\;\text{μs}$ (see section S4 for details). This agrees well with previous studies on the Fe-FePc complex (*23*) and exceeds relaxation times reported for conventional S = 1/2 systems on MgO/Ag(100) (*13*, *16*). We attribute the enhanced lifetime to the absence of tunneling through the remote spin and to the correlated ground state of the Fe-FePc complex (*23*). This highlights another crucial condition for electric spin pumping, as the spin lifetime must be sufficiently long to enable initialization before relaxation occurs.

## DISCUSSION

In this work, we demonstrate an all-electrical route to remotely control the state occupation of (molecular) electron spins, which offers a potential route toward more efficient electron spin initialization. We show that the concept of electrical hyperpolarization, previously established for nuclear spins of single atoms, can provide a route toward hyperpolarization of molecular electron spins. Using pairs of Fe-FePc complexes, we show that a spin-polarized tunneling current acting on one molecule can control the spin state occupation of the second, spatially separated molecule. The resulting nonequilibrium steady state is governed by the direction and magnitude of the tunneling current as well as the degree of its spin polarization. Here, angular momentum transfer is mediated by the exchange interaction between the molecules instead of hyperfine coupling. In contrast to the hyperfine coupling in nuclear spin systems, the exchange interaction can be tuned through molecular arrangement and chemical design. Its tunability provides a route to enhance the efficiency of the spin-pumping mechanism and to estimate the lifetime of the electron spin.

Our results identify three essential ingredients required for electric spin pumping: (i) a magnetic electrode is necessary to provide a spin-polarized current, which causes an imbalance between excitation and relaxation. (ii) In addition, a finite state mixing between the two molecular spins is required to mediate the angular momentum transfer to the remote spin, which proceeds via two mechanisms: The first involves an initial pumping of the sensing spin followed by an FF interaction with the remote spin. The second mechanism corresponds to a direct excitation of the remote spin. Both mechanisms can be efficiently tuned by the strength of the perpendicular exchange between the spins. (iii) Last, these inelastic tunneling rates must exceed the intrinsic relaxation to drive the system out of thermal equilibrium.

Our analysis shows that for high tunneling currents, the molecular spins adopt the spin polarization of the tip, which sets the limit to their effective polarization. Therefore, increasing the spin polarization of the tip is highly desirable. A recent study demonstrated a nearly fully spin-polarized tunneling current in a nickelocene-iron tunnel junction (*38*), which highlights the potential of engineered tunnel junctions for improved spin pumping. Another approach is the use of Yu-Shiba-Rusinov (YSR) states, which can be created by adding a magnetic impurity to a superconducting tip. This enables fully spin-polarized currents when tunneling through the YSR state (*39*, *40*).

Beyond the specific molecular spin system studied here, our findings demonstrate how remote spin pumping of individual electron spins can be achieved generally via electric currents. The proposed mechanism is supported by the simple model used to describe it. In this model, the spin system represented by an effective spin Hamiltonian is treated as an open quantum system coupled to two electrodes. Within this framework, the mechanism is not specific to the Fe-FePc complexes or the MgO/Ag(100) substrate used in this study. Other insulating layers could, in principle, serve the same role, provided that they decouple the spin effectively from scattering with substrate electrons to ensure a sufficiently long lifetime. We therefore expect the electrical spin pumping to be transferable to other spin systems such as atoms, solid-state spin defects, and different magnetic molecules including conjugated molecular systems. We further expect that it can be implemented in other device architectures in which a spin system is embedded in a double-barrier tunnel junction, such as semiconductor quantum dots or molecular break junctions.

The ability to induce spin polarization different from thermal equilibrium may also help to perform single spin ESR at higher temperatures above 1K (see section S5 and fig. S9) as realized in optically detected magnetic resonance (*41*). In addition, electron spin pumping can be extended to spin chains (see section S5 and fig. S10), which highlights its potential for larger spin systems. These capabilities are essential for scaling toward whole molecular spin networks, quantum simulators, and distributed quantum architectures.

## MATERIALS AND METHODS

### Experimental setup

The measurements were conducted in a Unisoku USM1600 scanning tunneling microscope mounted on a home-built dilution refrigerator operating at a base temperature of 50 mK. The DC bias voltage $V_{\text{DC}}$ and radio-frequency voltage $V_{\text{RF}}$ were applied to the tip. For the data evaluation, the sign of the stated $V_{\text{DC}}$ values was changed corresponding to the convention with bias applied to the sample. The microwave signal was generated by a Rohde & Schwarz SMB100B generator and mixed with $V_{\text{DC}}$ through a Microwave MDPX-0305 Diplexer from Marki. For the ESR measurements, magnetic tips were prepared by picking up several Fe atoms from the MgO surface. The used tips usually showed a step at $V_{\text{DC}}=0\;\text{mV}$ in differential conductance (d*I*/d*V*) measurements at the Fe site of the Fe-FePc molecules stemming from inelastic tunneling with a spin polarized tip (*31*). In addition, a magnetic field perpendicular to the sample surface was applied. For ESR frequency sweeps, we used a lock-in detection scheme where the RF voltage $V_{\text{RF}}$ was modulated at 323 Hz while the DC voltage $V_{\text{DC}}$ was applied continuously. For readout, we used a Stanford Research Systems SR860 digital lock-in amplifier.

### Sample preparation

The sample was prepared in situ under ultrahigh vacuum with a base pressure of <5 × 10^−10^ mbar. The Ag(100) crystal was cleaned by multiple cycles of argon ion sputtering and subsequent annealing. Afterward, magnesium oxide islands were grown by evaporating Mg in an oxygen-rich atmosphere for 10 min onto the heated silver crystal held at 510°C with an oxygen pressure of ≈1 × 10^−6^ mbar. The deposition resulted in a coverage of ≈50% with island thicknesses ranging from two to five monolayers. Via a home-built Knudsen cell, FePc molecules were deposited onto the surface with a deposition time of ≈90 s. For the subsequent Fe atom deposition, the sample was cooled down to ≈10 to 20 K. The Fe atoms were deposited for 21 s using e-beam evaporation leading to a sub-monolayer coverage.

## Supplementary Materials

### This PDF file includes:

Supplementary Text Figs. S1 to S10 Table S1 References
