# Supplementary material for: Electrical spin pumping in exchange-coupled molecules
Source: Sci Adv. 2026 Nov 13;12(46):eaei0428. doi: 10.1126/sciadv.aei0428 (PMC13763944; doi:10.1126/sciadv.aei0428)
Supplement: Supplementary file 1 [file sciadv.aei0428_sm.pdf]

Science Advances
AAAS

Supplementary Materials for

Electrical spin pumping in exchange-coupled molecules

Paul Greule *et al.*

Corresponding author: Philip Willke, philip.willke@kit.edu

Sci. Adv. **12**, eaei0428 (2026)
DOI: 10.1126/sciadv.aei0428

This PDF file includes:

Supplementary Text
Figs. S1 to S10
Table S1
References

Supplementary Text

Section S1. Spin Hamiltonian

We present here the spin Hamiltonian used in this work together with its solution as it forms the basis for deriving the transition matrix elements discussed in Supplementary Section S3 below as well as the experimental determination of the spin Hamiltonian parameters. As outlined in Ref. (*23*) Fe-FePc complexes can be modeled as effective S=1/2 systems. We therefore describe the molecule pairs consisting of two Fe-FePc complexes with the following Hamiltonian (*15, 23, 25, 26*) (Also see Eq. 1):

$$H = -g\mu_B\left(B + B_{\mathrm{tip}}\right)S_{\mathrm{A,z}} - g\mu_B B S_{\mathrm{B,z}} + J_\perp\left(S_{\mathrm{A,x}}S_{\mathrm{B,x}} + S_{\mathrm{A,y}}S_{\mathrm{B,y}}\right) + J_{||}S_{\mathrm{A,z}}S_{\mathrm{B,z}} \tag{S1}$$

With the respective Zeeman terms where the sensing spin S_{A} experiences an additional tip field B_{tip} to the external magnetic field B. For simplicity a transverse component of the tip field along the x- and y-direction is neglected because the tip field is small compared to the external field ($\approx$ 10 %) and a transverse component would only cause a rescaling of the tip spin polarization η. The exchange interaction is parametrized with the perpendicular $J_\perp$ and parallel interaction $J_{||}$ with respect to the quantization axis, given by the external magnetic field. We note, that the weaker dipole-dipole ($\approx$ 50 MHz) interaction is absorbed in the exchange parameters. g is the g-factor and μ_{B} the Bohr magneton. For $g\mu_{\mathrm{B}}B > J_{||}$ diagonalization of Eq. S1 yields four eigenstates which can be expressed as:

$$|0\rangle = |\uparrow\uparrow\rangle \tag{S2}$$

$$|1\rangle = \frac{-\alpha}{\sqrt{1+\alpha^2}}|\uparrow\downarrow\rangle + \frac{1}{\sqrt{1+\alpha^2}}|\downarrow\uparrow\rangle \tag{S3}$$

$$|2\rangle = \frac{1}{\sqrt{1+\alpha^2}}|\uparrow\downarrow\rangle + \frac{\alpha}{\sqrt{1+\alpha^2}}|\downarrow\uparrow\rangle \tag{S4}$$

$$|3\rangle = |\downarrow\downarrow\rangle \tag{S5}$$

With the parameter $\alpha = \frac{\delta+\sqrt{\delta^2+J_\perp^2}}{J_\perp}$ and the detuning $\delta = g\mu_{\mathrm{B}}B_{\mathrm{tip}}$ quantifying the difference of the Zeeman energies of the two individual spins. The eigenenergies of the spin system are given by:

$$E_0 = \frac{1}{4}\left(-2g\mu_\mathrm{B}B_\mathrm{tip} - 4g\mu_\mathrm{B}B + J_\parallel\right) \tag{S6}$$

$$E_1 = \frac{1}{4}\left(-J_\parallel - 2\sqrt{\delta^2 + J_\perp^2}\right) \tag{S7}$$

$$E_2 = \frac{1}{4}\left(-J_\parallel + 2\sqrt{\delta^2 + J_\perp^2}\right) \tag{S8}$$

$$E_3 = \frac{1}{4}\left(2g\mu_\mathrm{B}B_\mathrm{tip} + 4g\mu_\mathrm{B}B + J_\parallel\right) \tag{S9}$$

A corresponding energy level diagram is displayed in Fig. S2A. Generally, four ESR transitions with $\Delta m = \pm 1$ are allowed: $f_\mathrm{I} = E_3 - E_2$, $f_\mathrm{II} = E_1 - E_0$, $f_\mathrm{III} = E_3 - E_1$ and $f_\mathrm{IV} = E_2 - E_0$. We note that we put the Planck constant $h = 1$. The resonance frequencies read:

$$f_\mathrm{I} = \frac{1}{2}\left(2g\mu_\mathrm{B}B + g\mu_\mathrm{B}B_\mathrm{tip} + J_\parallel - \sqrt{\delta^2 + J_\perp^2}\right) \tag{S10}$$

$$f_\mathrm{II} = \frac{1}{2}\left(2g\mu_\mathrm{B}B + g\mu_\mathrm{B}B_\mathrm{tip} - J_\parallel - \sqrt{\delta^2 + J_\perp^2}\right) \tag{S11}$$

$$f_\mathrm{III} = \frac{1}{2}\left(2g\mu_\mathrm{B}B + g\mu_\mathrm{B}B_\mathrm{tip} + J_\parallel + \sqrt{\delta^2 + J_\perp^2}\right) \tag{S12}$$

$$f_\mathrm{IV} = \frac{1}{2}\left(2g\mu_\mathrm{B}B + g\mu_\mathrm{B}B_\mathrm{tip} - J_\parallel + \sqrt{\delta^2 + J_\perp^2}\right) \tag{S13}$$

Zero Detuning

For $\delta = 0$ all four transitions (f_I-f_IV) are allowed when performing ESR on the sensing spin $\mathrm{S_A}$ caused by the state mixing (see Fig. S2B top). From the resonance frequencies the exchange interaction parameters can be determined: $|J_\perp| = f_\mathrm{III} - f_\mathrm{I} = f_\mathrm{IV} - f_\mathrm{II}$ and $J_\parallel = f_\mathrm{I} - f_\mathrm{II} = f_\mathrm{III} - f_\mathrm{IV}$. The sign of $J_\parallel$ further reveals whether the exchange is ferromagnetic $J_\parallel < 0$ or antiferromagnetic $J_\parallel > 0$. Here, one must be able to assign the observed resonance peaks to the respective transitions. If the systems state occupation is governed by the thermal Boltzmann distribution, this can be achieved by the relative peak amplitudes. For ferromagnetic coupling the resonances corresponding to f_I and f_III are less pronounced than f_II and f_IV because they involve eigenstates with higher energy which

are less occupied. In contrast, for antiferromagnetic coupling f_{I} and f_{III} are more pronounced than f_{II} and f_{IV} (see Fig. S2B). These relations allow to determine the coupling type and the magnitude of $J_{\perp}$ and $J_{\parallel}$. Table S1 presents the determined parameters for the complex pairs presented in Fig. 4. We note that in order to provide a solid analysis one must be careful with the assignment of the resonance peaks when spin pumping is present. In this case one must operate at low currents or in the regime where spin relaxation is enhanced to assure that the state occupations are not inverted.

Detuned Regime

For the detuned regime, where $B_{\mathrm{tip}} > 0$ and thus $\delta > 0$ as well as $\delta > |J_{\perp}|$, the eigenstates are well presented by the Zeeman product states having $|1\rangle \approx |\uparrow\downarrow\rangle$ and $|2\rangle \approx |\downarrow\uparrow\rangle$. In this regime the voltage-dependent frequency sweeps presented in this work are performed. Here, only the two transitions corresponding to f_{III} and f_{IV} are allowed (see Fig. S2B bottom). The detuning δ is obtained from the relative frequency shift with respect to the avoided level crossing. An experimental example is shown in Fig. S3. Knowing the coupling type from the evaluation strategy described above we can determine the state occupation. For ferromagnetic coupling $J_{\parallel} < 0$ we find the occupation of the remote spin's ground state $|\mathrm{g}\rangle$ to correspond to the resonance peak at higher frequency. For the antiferromagnetic case $J_{\parallel} > 0$ we find the occupation of $|\mathrm{g}\rangle$ to correspond to the resonance peak at lower frequency.

Section S2. Rate Simulation

To model the state occupation of the spin system under the influence of the tunneling current we describe the surface spins as an open quantum system interacting with the electronic reservoirs (*31, 34, 35*). Within this framework, we assume only a weak interaction of the spins with their environment, corresponding to the Born approximation. The eigenstates and eigenenergies of the spin system are therefore determined by the effective spin Hamiltonian (see Supplementary Section S1). The coupling to the environment induces transitions between these eigenstates, with the occupation p_i denoting the probability of finding the system in the state $|\psi_i\rangle$. Furthermore, the electronic reservoirs are assumed to be much larger than the spin system and are therefore unaffected by its dynamics.

In the simulation, the interaction with the tunneling electrons is described via second order scattering rates Γ (co-tunneling). First order scattering (sequential tunneling) is exponentially suppressed because the Coulomb repulsion energy is larger than the applied bias voltage. The system is therefore operated in the Coulomb blockade regime. As mentioned in the main text, we model the tip (t) and sample (s) electrodes by two electronic reservoirs with $n^t_\uparrow = \frac{1+\eta}{2}$, $n^t_\downarrow = \frac{1-\eta}{2}$ and $n^s_\uparrow = n^s_\downarrow = \frac{1}{2}$ with the spin polarization of the tip η. Here, the Fermi-Dirac distributions of the two electrodes are thermally broadened by the temperature T. The simulation code is available at Ref. (*29*). In our model, the sensing spin S_A experiences tunneling rates from tip and sample $\Gamma^{s\to t}_A$, $\Gamma^{t\to s}_A$, $\Gamma^{t\to t}_A$ and $\Gamma^{s\to s}_A$. The remote spin S_B which is distant from the tip electrode only experiences scattering with electrons from the surface $\Gamma^{s\to s}_B$. A schematic drawing of the tunneling processes is shown in Fig. S4. We note here, that the transition rates induced by the ESR detection are small compared to the excitation and relaxation rates from electron scattering (*19*).

According to Ref. (*30–33*) the tunneling rates from state i to f of the spin system are given by:

$$\Gamma^{s\to t}_{A,if} = \frac{G_{st}}{e^2} P^{s\to t}_{A,if} \int_{-\infty}^{\infty} f(\epsilon + eV_{DC}, T)[1 - f(\epsilon - E_{if}, T)]d\epsilon \tag{S14}$$

$$\Gamma^{t\to s}_{A,if} = \frac{G_{st}}{e^2} P^{t\to s}_{A,if} \int_{-\infty}^{\infty} f(\epsilon - eV_{DC}, T)[1 - f(\epsilon - E_{if}, T)]d\epsilon \tag{S15}$$

$$\Gamma^{t\rightarrow t}_{\mathrm{A},if} = \frac{G_{tt}}{e^2} P^{t\rightarrow t}_{\mathrm{A},if} \int_{-\infty}^{\infty} f(\epsilon, T)[1 - f(\epsilon - E_{if}, T)] d\epsilon \tag{S16}$$

$$\Gamma^{s\rightarrow s}_{\mathrm{A},if} = \frac{G_{ss}}{e^2} P^{s\rightarrow s}_{\mathrm{A},if} \int_{-\infty}^{\infty} f(\epsilon, T)[1 - f(\epsilon - E_{if}, T)] d\epsilon \tag{S17}$$

$$\Gamma^{s\rightarrow s}_{\mathrm{B},if} = \frac{G_{ss}}{e^2} P^{s\rightarrow s}_{\mathrm{B},if} \int_{-\infty}^{\infty} f(\epsilon, T)[1 - f(\epsilon - E_{if}, T)] d\epsilon \tag{S18}$$

Here, e is the electron charge, G_{st} the zero voltage conductance of the tunnel junction, G_{ss} the sample-sample conductance and G_{tt} the tip-tip conductance. $E_{if} = E_f - E_i$ is the energy difference of the involved states (see Eq. S6-S9). $P_{\mathrm{A},if}$ and $P_{\mathrm{B},if}$ are scattering probabilities based on the matrix elements between electrons with spin $\mathrm{S_A}$ and spin $\mathrm{S_B}$ as described in Ref. (*30*).

The state occupation p_i is then given by the steady state solution of the rate equation ($t \rightarrow \infty$):

$$\frac{d}{dt} p_i(t) = \sum_{f \neq i} p_f(t) \Gamma_{fi} - p_i(t) \sum_{f \neq i} \Gamma_{if} \tag{S19}$$

With $\Gamma_{if} = \Gamma^{t\rightarrow s}_{\mathrm{A},if} + \Gamma^{s\rightarrow t}_{\mathrm{A},if} + \Gamma^{t\rightarrow t}_{\mathrm{A},if} + \Gamma^{s\rightarrow s}_{\mathrm{A},if} + \Gamma^{s\rightarrow s}_{\mathrm{B},if}$ the sum of all rates.

Section S3. Analytical Approximation

In this section, we derive an analytical solution of the rate equation (Eq. S19) for the spin system described in Supplementary Section S1. Throughout the derivation we make several approximations that are justified by the experimental operating regime: Since the applied voltages are much larger than the eigenenergies of the spin system as well as the thermal energy, $|eV_{\mathrm{DC}}| > E_i > k_{\mathrm{B}}T$, the dynamics of the sensing spin is dominated by sample-tip or tip-sample tunneling events. Consequently, the sensing spin is immediately polarized by the tunneling current and can be treated as having a voltage independent magnetization. This allows us to determine the magnetization of the remote spin in two steps:

We first compute the state occupation of the sensor spin S_{A} and then in a second step the occupation of the remote spin S_{B}. For computing the rates of the individual S=1/2 spins, we use the rate equation of a two-level system which reads as:

$$\frac{d}{dt}\begin{pmatrix} p_{\mathrm{g}} \\ p_{\mathrm{e}} \end{pmatrix} = \begin{pmatrix} -\Gamma_{\mathrm{exc}} & \Gamma_{\mathrm{relax}} \\ \Gamma_{\mathrm{exc}} & -\Gamma_{\mathrm{relax}} \end{pmatrix} \begin{pmatrix} p_{\mathrm{g}} \\ p_{\mathrm{e}} \end{pmatrix} \tag{S20}$$

With the occupation of the ground (excited) state p_{g} (p_{e}) and the sum of all rates that excite (relax) the spin Γ_{exc} (Γ_{relax}). We find the steady state solution for $t \longrightarrow \infty$: $p_{\mathrm{g}} = \frac{\Gamma_{\mathrm{relax}}}{\Gamma_{\mathrm{exc}}+\Gamma_{\mathrm{relax}}}$ and $p_{\mathrm{e}} = \frac{\Gamma_{\mathrm{exc}}}{\Gamma_{\mathrm{exc}}+\Gamma_{\mathrm{relax}}}$ and consequently the magnetization $M = \frac{\Gamma_{\mathrm{relax}}-\Gamma_{\mathrm{exc}}}{\Gamma_{\mathrm{relax}}+\Gamma_{\mathrm{exc}}}$.

Sensing Spin

As mentioned above, for S_{A} the rates from tip to sample and sample to tip are dominant, since the potential difference is the dominant energy scale $|eV_{\mathrm{DC}}| > E_i > k_{\mathrm{B}}T$ while the conductances are of similar magnitude $G_{ss} \approx G_{tt} \approx G_{st}$. Therefore, we can neglect the inelastic transitions from surface-surface or tip-tip processes. We also neglect the finite terms that include scattering with the remote spin due to the low mixing of the spin states in the detuned regime. The excitation and relaxation rates of the sensing spin are then given by:

$$\Gamma_{\mathrm{exc}} = \frac{G_{st}}{e} |V_{\mathrm{DC}}| \frac{1}{2} \frac{1 \mp \eta}{2} \tag{S21}$$

$$\Gamma_{\mathrm{relax}} = \frac{G_{st}}{e} |V_{\mathrm{DC}}| \frac{1}{2} \frac{1 \pm \eta}{2} \tag{S22}$$

where the upper (lower) sign applies to $V_{DC} > 0$ ($V_{DC} < 0$). For $V_{DC} > 0$ we obtain $p_{A,g} = \frac{1+\eta}{2}$, $M_A = \eta$ and for $V_{DC} < 0$: $p_{A,g} = \frac{1-\eta}{2}$, $M_A = -\eta$. Thus, S_A fully adopts the spin polarization of the magnetic tip: Depending on the voltage polarity, it is either driven into the ground or excited state.

Remote Spin

For S_B we consider the relaxation and excitation from the tip-sample processes with S_A which have a finite probabilities to scatter S_B caused by the small state mixing. We describe the rates by multiplying the transition matrix element of the combined system with the respective occupation of S_A described in the section above. In addition, we include the relaxation process from sample-sample scattering as the next most relevant contribution:

$$\Gamma_{\mathrm{exc}} = \frac{G_{st}}{e}|V_{\mathrm{DC}}|\Big(p_{\mathrm{A,g}}P_{\mathrm{A,01}} + p_{\mathrm{A,e}}P_{\mathrm{A,21}} + p_{\mathrm{A,e}}P_{\mathrm{A,23}}\Big) \tag{S23}$$

$$\begin{aligned}\Gamma_{\mathrm{relax}} = &\frac{G_{st}}{e}\,|V_{\mathrm{DC}}|\Big(p_{\mathrm{A,g}}P_{\mathrm{A,10}} + p_{\mathrm{A,g}}P_{\mathrm{A,12}} + p_{\mathrm{A,e}}P_{\mathrm{A,32}}\Big)\\ &+ \frac{G_{ss}}{e^2}\,E_{10}\Big(p_{\mathrm{A,g}}P^{s\to s}_{\mathrm{B,10}} + p_{\mathrm{A,e}}P^{s\to s}_{\mathrm{B,32}}\Big)\end{aligned} \tag{S24}$$

Here, the matrix elements $P_{\mathrm{A},if}$ are given by $P^{t\to s}_{\mathrm{A},if}$ ($P^{s\to t}_{\mathrm{A},if}$) for $V_{DC} > 0$ ($V_{DC} < 0$). In the descriptions of Eq. S23 and S24 we identify two processes which when tunneling through the sensing spin excite or relax the remote spin (Fig. S5). One is a flip-flop interaction (FF) between S_A and S_B quantified by $P_{\mathrm{A,21}}$ and $P_{\mathrm{A,12}}$. The other is a direct scattering of S_B quantified by $P_{\mathrm{A,01}}$ ($P_{\mathrm{A,10}}$) and $P_{\mathrm{A,23}}$ ($P_{\mathrm{A,32}}$) referred to as remote scattering (RS).

For the eigenstates found in Supplementary Section S1 we can compute the relevant transition probabilities based on the matrix elements:

$$P^{s\to t}_{\mathrm{A,01}} = P^{s\to t}_{\mathrm{A,23}} = P^{t\to s}_{\mathrm{A,10}} = P^{t\to s}_{\mathrm{A,32}} = \frac{1}{2}\frac{1+\eta}{2}\frac{1}{1+\alpha^2} \tag{S25}$$

$$P^{s\to t}_{\mathrm{A,10}} = P^{s\to t}_{\mathrm{A,32}} = P^{t\to s}_{\mathrm{A,01}} = P^{t\to s}_{\mathrm{A,23}} = \frac{1}{2}\frac{1-\eta}{2}\frac{1}{1+\alpha^2} \tag{S26}$$

$$P^{s\to t}_{\mathrm{A,21}} = P^{s\to t}_{\mathrm{A,12}} = P^{t\to s}_{\mathrm{A,21}} = P^{t\to s}_{\mathrm{A,12}} = \frac{1}{2}(\frac{\alpha}{1+\alpha^2})^2 \tag{S27}$$

$$P^{s\to s}_{\mathrm{B},10} = P^{s\to s}_{\mathrm{B},32} = \frac{1}{4} \tag{S28}$$

Here, we identify the transition matrix element for the flip-flop process $\mathcal{M}_{\mathrm{FF}} = (\frac{\alpha}{1+\alpha^2})^2 = \frac{J_\perp^2}{4(\delta^2+J_\perp^2)}$ and the remote scattering process $\mathcal{M}_{\mathrm{RS}} = \frac{1}{1+\alpha^2} = \frac{1}{2} - \frac{\delta}{2\sqrt{\delta^2+J_\perp^2}}$ presented in Eq. 2 and Eq. 3, respectively. They depend on the detuning δ and exchange coupling $J_\perp$ of the two spins. We note that we simplified $E_{10} = E_{32} \approx g\mu_{\mathrm{B}}B$, since $|J_{\|}| < g\mu_{\mathrm{B}}B_{\mathrm{tip}} < g\mu_{\mathrm{B}}B$.
For the magnetization of $\mathrm{S_B}$ we then get:

$$M_{\mathrm{B}} = \frac{\eta V_{\mathrm{DC}} G_{st}\mathcal{M} + g\mu_{\mathrm{B}}B\, G_{ss}/2e}{|V_{\mathrm{DC}}| G_{st}\mathcal{M} + g\mu_{\mathrm{B}}B\, G_{ss}/2e} \tag{S29}$$

With $\mathcal{M} = \mathcal{M}_{\mathrm{RS}} + \mathcal{M}_{\mathrm{FF}}$. For $G_{\mathrm{set}} \approx G_{st}$, where $G_{\mathrm{set}} = I/V_{\mathrm{DC}}$ is the setpoint conductance in the STM experiment, we can express M_{B} as a function of the induced tunneling current $I = G_{st}V_{\mathrm{DC}}$ which is independent of G_{set} and arrive at the formula given in the main text (Eq. 4):

$$M_{\mathrm{B}}(I) = \eta\frac{I - I_0}{|I| + |\eta I_0|} \quad \text{with} \quad I_0 = -\frac{G_{ss} g\mu_{\mathrm{B}}B}{2e\mathcal{M}\eta} \tag{S30}$$

With the characteristic tunneling current I_0 describing the current needed to pump the system to $M_{\mathrm{B}} = 0$.

In the limit of vanishing tunneling current for $V_{\mathrm{DC}} \to 0$, the occupation of the spin states is governed primarily by their intrinsic energy splitting rather than by current-induced transitions and Eq. 4 is no longer valid. Instead, the state occupations approach thermal equilibrium which is determined by the temperature T entering the Fermi–Dirac distributions of the electronic reservoirs. Measurements performed at small V_{DC} therefore provide an upper bound of the spin temperature in the absence of tunneling current. For the experimental data presented in this work, this spin temperature was determined to be $T = 0.2$ K from the numerical solution of the full rate equation presented in Supplementary Section S2.

Section S4. Lifetime Estimation

Since the pumping of the remote spin depends on the ratio between excitation via the spin-polarized tunneling current and relaxation via substrate electrons, one can estimate the lifetime of the remote spin in the absence of any pumping. This lifetime is given by:

$$1/T_1 = \Gamma_{\mathrm{B}}^{s \to s} = \frac{G_{ss}}{e^2} E_{10} \cdot P \tag{S31}$$

Where P is the probability of an inelastic excitation. With the characteristic current $I_0 = -\frac{G_{ss} g \mu_{\mathrm{B}} B}{2e\mathcal{M}\eta}$ we find:

$$-I_0 \eta = \frac{e}{2P \cdot T_1} \frac{1}{\mathcal{M}} \tag{S32}$$

where we have used $E_{10} = g\mu_{\mathrm{B}} B$. For the four investigated complex pairs presented in Fig. 4, which exhibit different $\mathcal{M}$ values, we can plot this relation as shown in Fig. S8. Using $P \approx 17$ %, which was determined for the Fe-FePc complex in previous work (*23*), we find that our measurements exhibit a spin lifetime in the range of 1-10 μs.

Section S5. Additional Simulations

Beyond the operating regime discussed above, in which the angular momentum is transferred within a spin pair at low temperatures, the employed model also allows to make predictions for other parameter regimes.

Elevated Temperatures

The spin-polarized tunneling current could, in principle, enable spin pumping at elevated temperatures. This is particularly relevant for single-spin ESR, where the detection relies on the spin contrast, i.e., the occupation difference of the spin states.

Using the full rate equation model presented in Supplementary Section S2, we simulated the magnetization of the remote spin as a function of tunneling current for temperatures ranging from 1–300 K, while keeping all other parameters comparable to those used in the present study. The temperature enters through the Fermi–Dirac distributions which describe the electronic reservoirs. With increasing temperature, the Fermi edges of the substrate and tip electrodes become broadened which increases the number of occupied states above E_F and unoccupied states below E_F. Consequently, inelastic electron-scattering processes that drive the system towards thermal equilibrium are enhanced, see Ref. (*22*) Eq. (S36). The tunneling-induced spin pumping competes with these processes which increases the required spin-polarized tunneling current to polarize the spin system. The results are shown in Fig. S9A. The curves illustrate how the remote spin is driven from thermal equilibrium occupation, given by the intercept at zero current, toward an increased ground state occupation, corresponding to positive M_B. To quantify the behavior, we define $I_{1/2}$ as the tunneling current required to reach the midpoint between the value at zero current and the tip spin polarization: $M_B(I_{1/2}) = M_B(0) + \frac{1}{2}[\eta - M_B(0)]$. As shown in Fig. S9B, $I_{1/2}$ increases linearly with temperature. This trend arises because scattering with substrate electrons is enhanced at higher temperatures, requiring a larger current to drive the spin out of thermal equilibrium. Interestingly, the simulation suggests that for sufficiently large currents in the nA regime the remote spin can be even polarized at room temperature. This demonstrates that electrical spin pumping can, in principle, serve as an initialization method to perform ESR at higher temperatures.

We note that this analysis is based on theoretical considerations only and that experimental im-

plementation at room temperature remains challenging. For instance, it requires an electrode (tip) that remains spin-polarized at room temperature. This has been shown for instance for STM tips fabricated from bulk magnetic materials, such as CrO_2 (*43*). Additionally, the molecules must remain stable inside the tunnel junction, which is more difficult with increasing thermal diffusion. One possible solution is to embed the molecule into a molecular monolayer.

Three spin chain

In principle, the described transfer of angular momentum is not limited to a pair of spins but can also be extended to larger coupled spin structures. To illustrate this, we consider a three spin chain in which an additional spin is coupled to the remote spin. In this model, the Zeeman energies are detuned by assigning different g-factors to the three spins instead of using a magnetic tip field. Now, the angular momentum is transferred from the tip which is acting on the first spin to the second and third spin. The resulting magnetization of each spin as a function of tunneling current is shown in Fig S10. The simulations demonstrate that more distant spins can also be polarized, but require progressively larger tunneling currents.

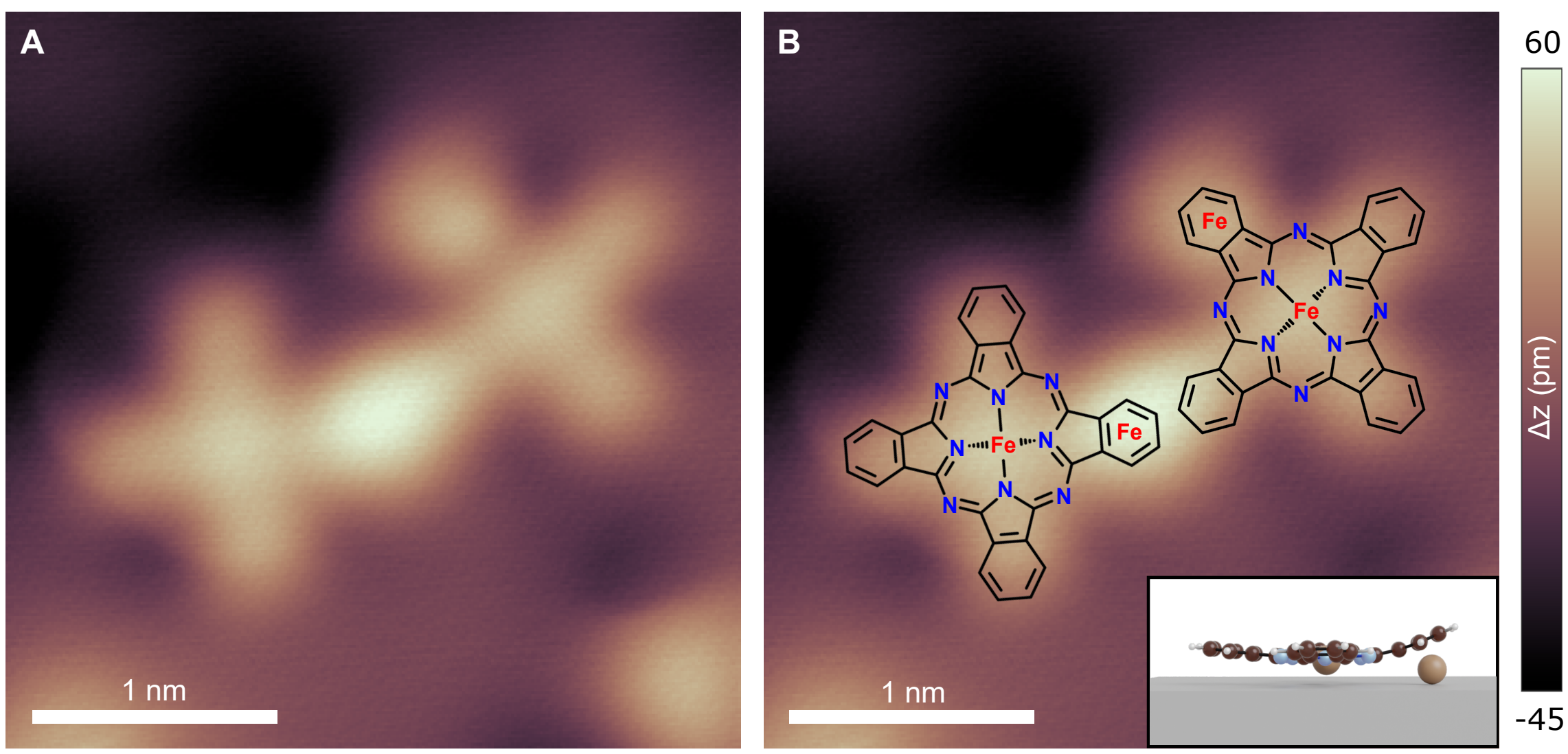

Figure S1: **Orientation of the Fe-FePc complex pair.** (**A**) STM topography of the Fe-FePc complex pair shown in Fig. 1B. (**B**) Same STM topography as in (A), with the chemical structures of the two complexes overlaid to indicate their relative orientation and the position of the Fe atoms. The inset shows a side view of the Fe-FePc molecular structure, illustrating that the additional Fe atom is located beneath one of the benzene rings.

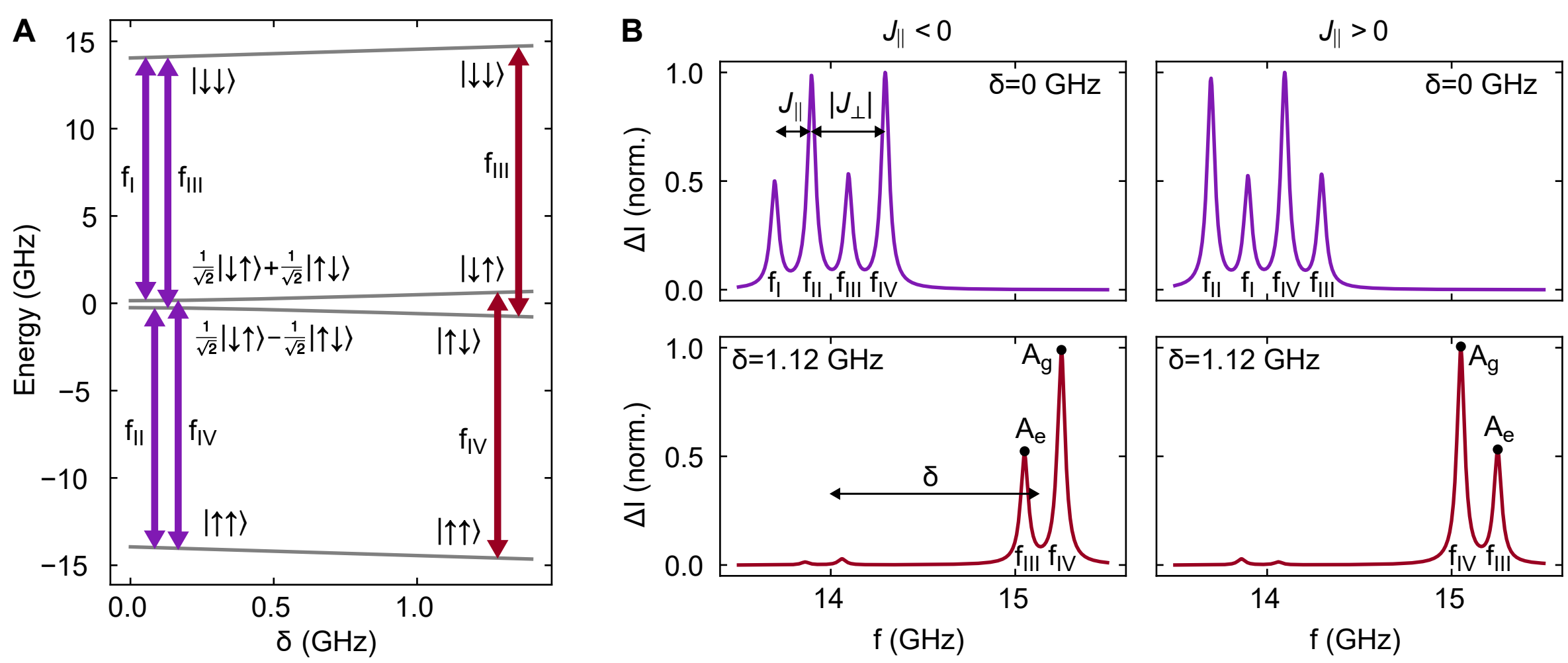

Figure S2: **Simulated ESR spectra.** (**A**) Energy level diagram of the coupled spin system as a function of detuning δ. Violet arrows illustrate the four possible transitions at the avoided level crossing $\delta = 0$. Red arrows illustrate the two remaining transitions in the detuned regime ($\delta > 0$). (**B**) ESR simulations (ΔI over f) for ferromagnetic (left) and antiferromagnetic (right) exchange coupling in the two regimes ($\delta = 0, \delta > 0$). Through the resonance peaks the exchange interaction parameters $|J_\perp|$ and $J_\parallel$, the state occupations $p_g \propto A_g, p_e \propto A_e$ and detuning δ can be obtained. [Simulation parameters: $B = 500$ mT, $g = 2$, $|J_\perp| = 400$ MHz, $J_\parallel = 200$ MHz, $T = 1$ K]

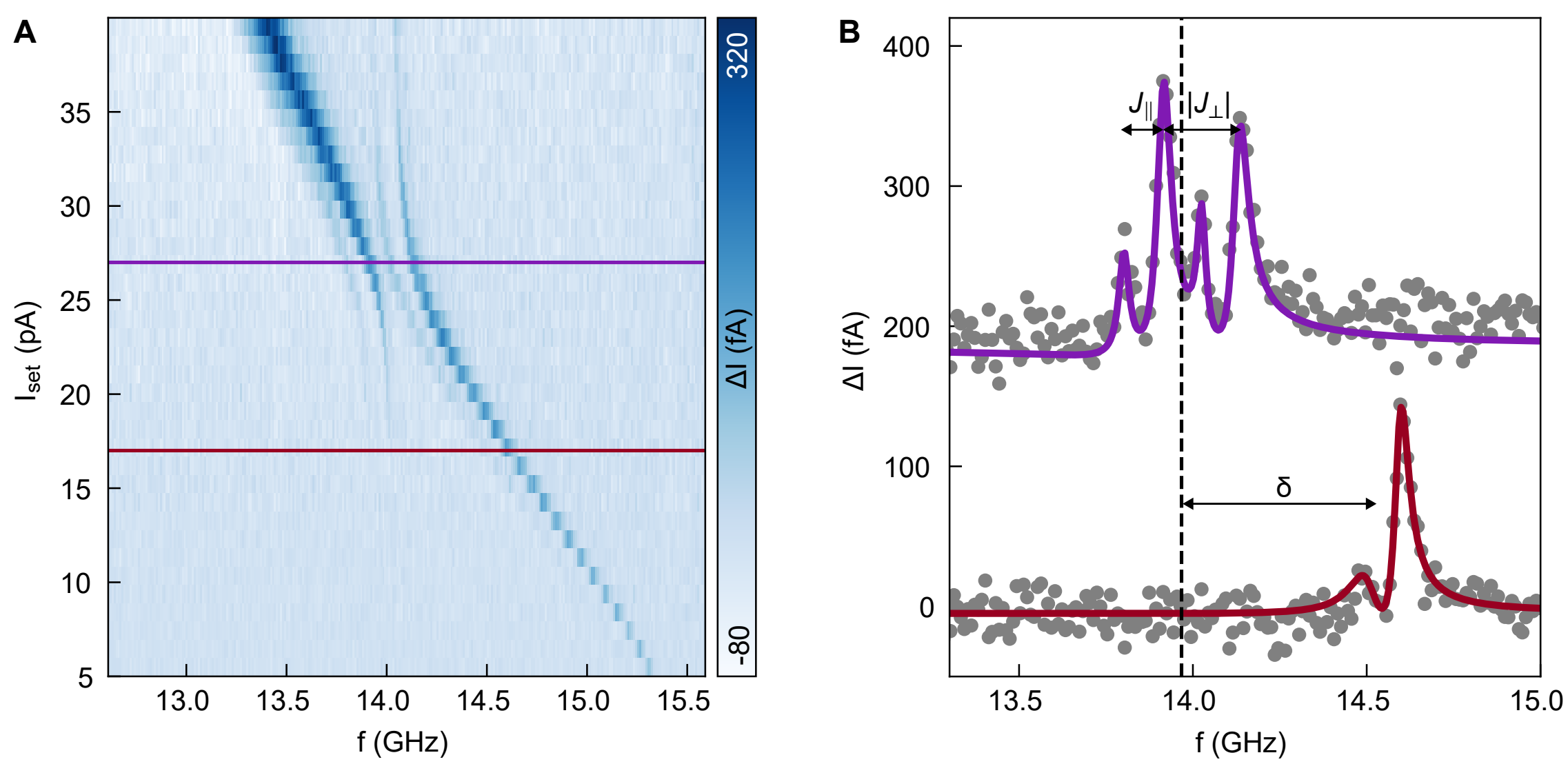

Figure S3: **Extraction of coupling parameters.** (**A**) Colormap of a ESR frequency sweep measured on a ferromagnetically coupled molecule pair showing the change in tunneling current ΔI as a function of radio frequency f and setpoint current I_{set}. As the setpoint is changed, the systems energy levels are tuned by the magnetic tip field through the avoided level crossing at which $\delta = 0$. The spectrum evolves from two to four and back to two resonance peaks, reflecting the hybridization of the spins close to $\delta = 0$. Solid horizontal lines indicate the traces displayed in (B) [ESR conditions: $V_{\text{DC}} = 40$ mV, $B = 494$ mT, $V_{\text{RF}} = 10$ mV]. (**B**) Single spectra showing ΔI over f. The upper trace (violet) shows the ESR signal measured at $\delta = 0$ from which the in-plane and out-of-plane exchange interactions, $|J_\perp|$ and $J_{||}$, are extracted (black arrows). The lower trace (red) exemplarily shows a frequency sweep in the detuned regime. The detuning δ is determined from the frequency shift relative to the avoided level crossing. The traces are vertically offset for clarity.

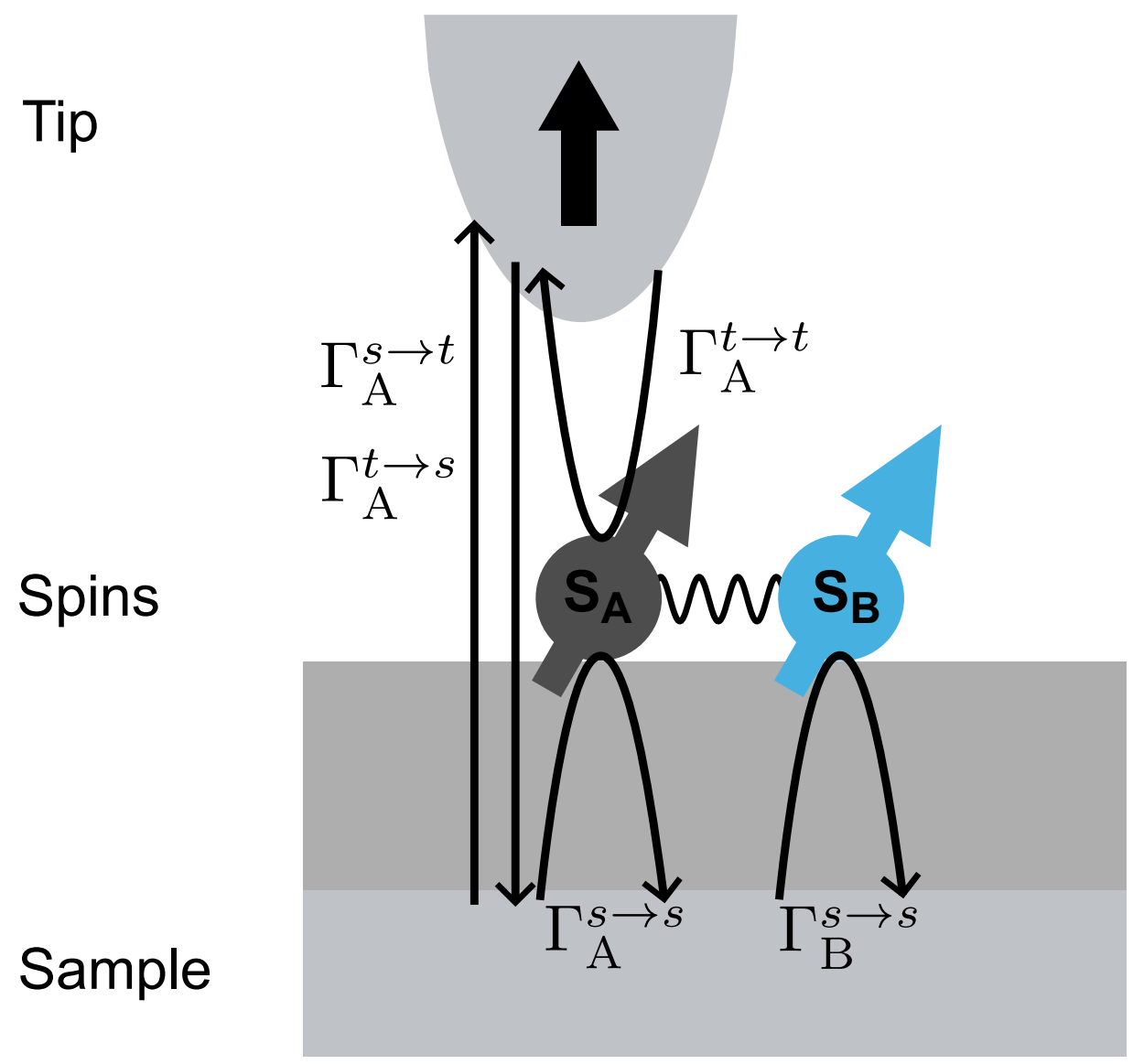

Figure S4: **Schematic of the tunnel junction geometry.** The image shows the tunneling processes considered in the full rate simulation. The surface spins are separated from the tip and sample electrodes by the vacuum barrier and the insulating MgO layer. Both spins undergo elastic and inelastic scattering with sample electrons. The sensing spin S_A, located below the spin-polarized tip, additionally couples to tip electrons. This enables additional tip-tip as well as tip-sample and sample-tip tunneling processes that allow the control over relaxation and excitation.

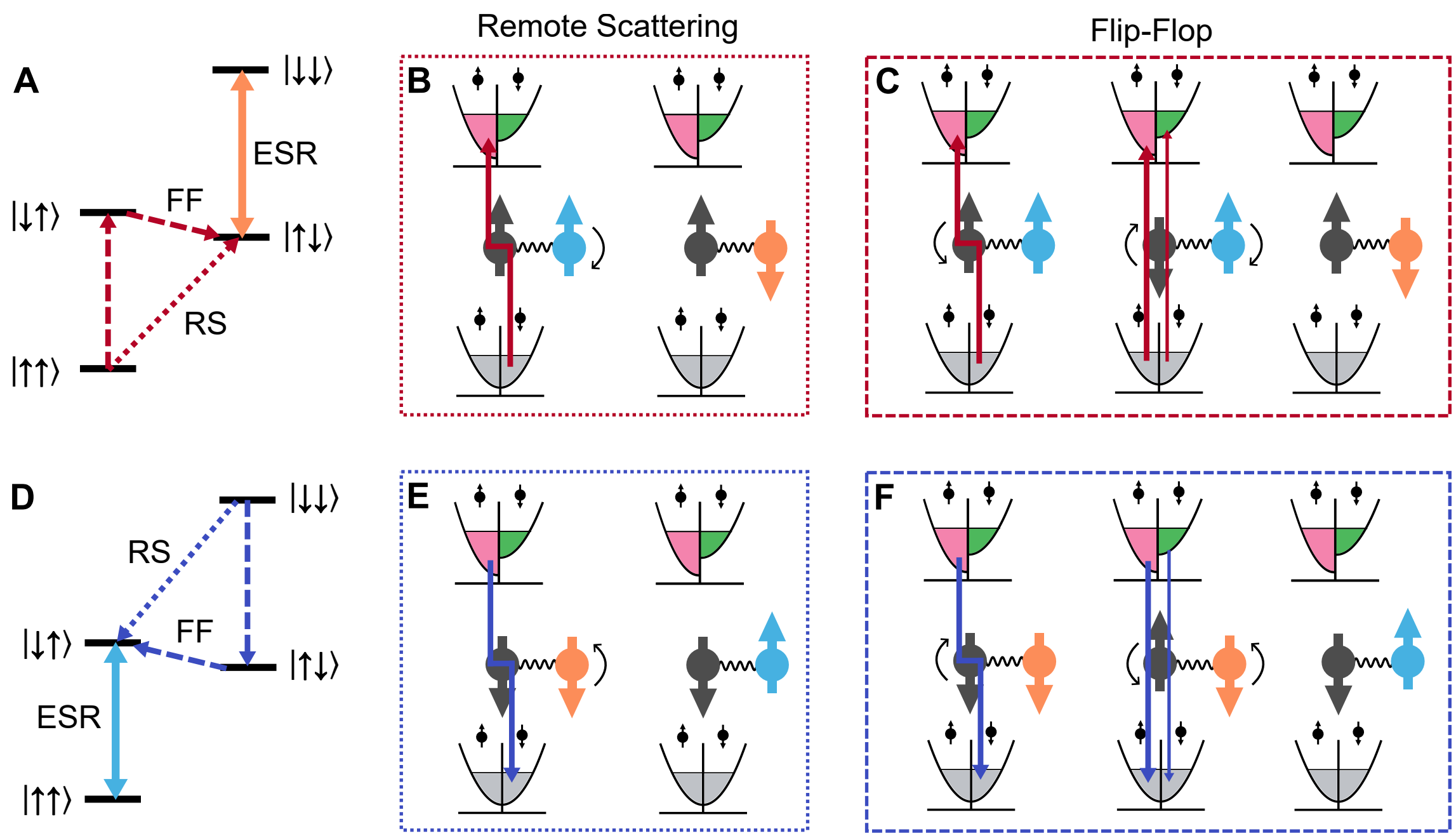

Figure S5: **Remote Scattering and Flip-Flop process.** (**A**) Energy level diagram with the remote scattering process (red dotted line), the flip-flop process (red dashed line) and the ESR detection (orange) indicated with arrows for current from sample to tip ($I < 0$). (**B**) Schematic of the inelastic scattering in the tunnel junction for the remote scattering. The tip is modeled via a spin-polarized electron bath ($\eta > 0$) with a higher density of states for up electrons (pink) than down electrons (green). In contrast, the sample electron bath is spin averaged. An electron tunnels from sample to tip transferring its angular momentum directly onto the remote spin causing it to be spin pumped into the excited state. (**C**) Schematic of the flip-flop interaction. First an electron excites the sensing spin, then a second electron mediates the flip-flop process between the sensing and remote spin. (**D-F**) The drawings show the respective processes discussed in (A-C) for the tunneling current flowing from tip to sample ($I > 0$) which causes the remote spin to relax.

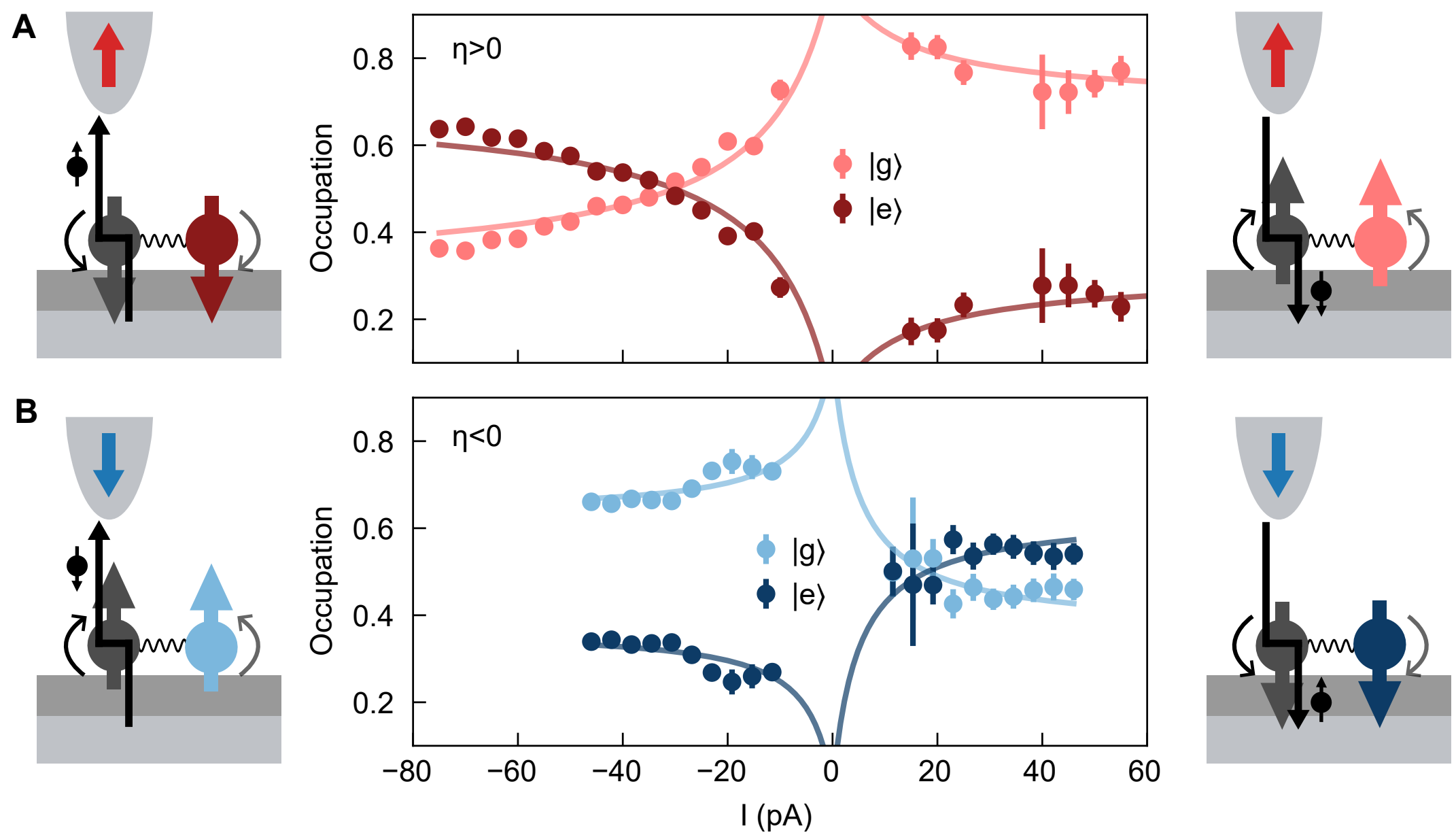

Figure S6: **State occupation for opposite tip polarizations.** (**A**) State occupations as a function of I for a positive tip polarization $\eta > 0$ [ESR conditions: $G_{\mathrm{set}} = 0.5$ nS, $B = 450$ mT, $V_{\mathrm{RF}} = 12$ mV]. The solid lines are fits to the experimental data (dots) based on Eq. 4 [fit results: $\eta = 0.38 \pm 0.02$, $I_0 = -15.0 \pm 1.0$ pA]. The sketch to the left (right) illustrates the spin excitation (relaxation) caused by the spin-polarized tunneling current for $\eta > 0$. (**B**) State occupations as a function of I for $\eta < 0$ [ESR conditions: $G_{\mathrm{set}} = 0.77$ nS, $B = 450$ mT, $V_{\mathrm{RF}} = 8$ mV, fit results: $\eta = -0.27 \pm 0.02$, $I_0 = 18.1 \pm 1.7$ pA].

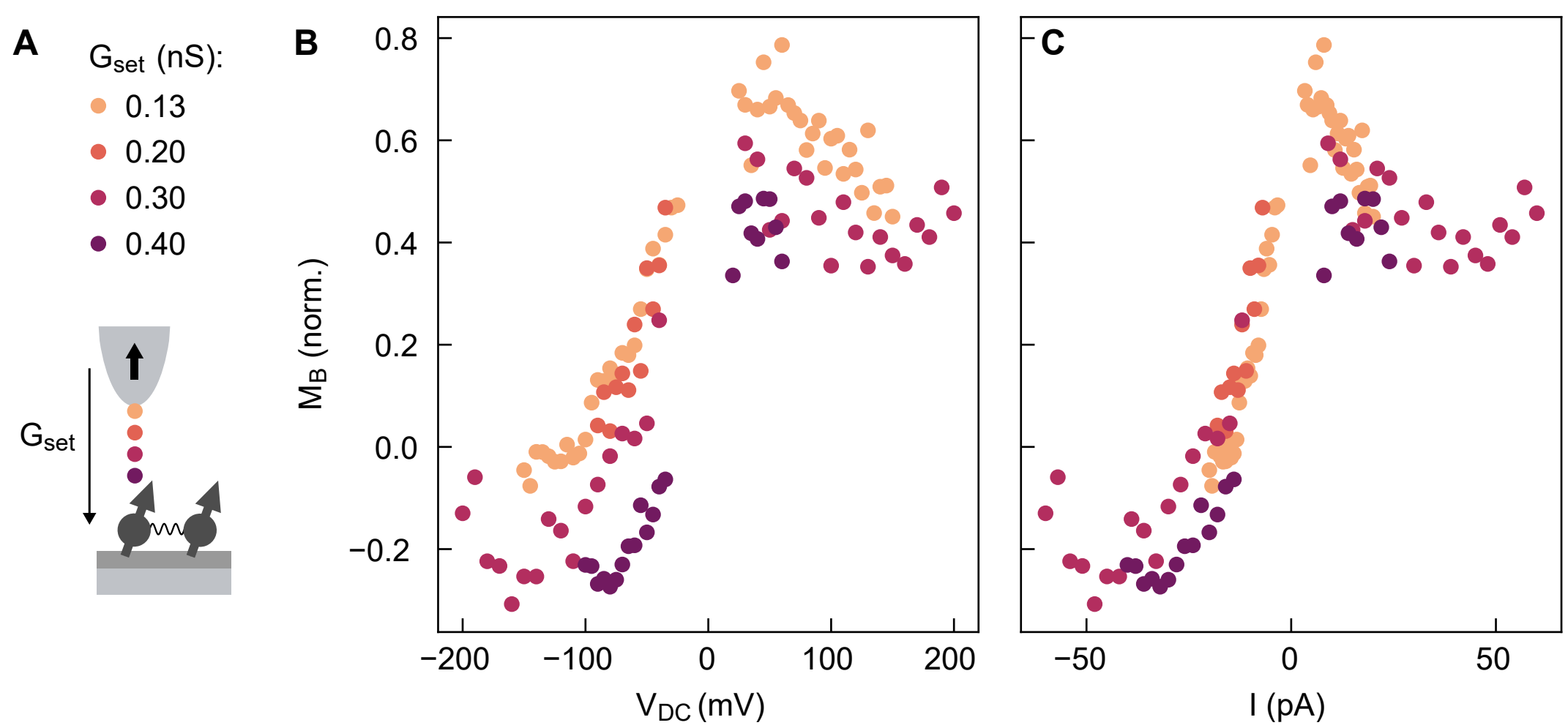

Figure S7: **Setpoint dependence.** (**A**) Schematic of the tunnel junction illustrating how the STM tip is positioned closer to the surface when increasing the setpoint conductance $G_{set} = I_{set}/V_{set}$. (**B**) Magnetization of the remote spin M_B as a function of bias voltage V_{DC} for different G_{set} measured on the same molecule pair. Increasing G_{set} enhances the spin pumping efficiency, reflected by a steeper evolution of M_B with bias. [ESR conditions: G_{set} = 0.13 nS, B = 458 mT, V_{RF} = 10 mV, G_{set} = 0.2 nS, B = 458 mT, V_{RF} = 10 mV, G_{set} = 0.3 nS, B = 494 mT, V_{RF} = 10 mV, G_{set} = 0.4 nS, B = 484 mT, V_{RF} = 12 mV]. (**C**) Same data as in (B) showing M_B over $I = G_{set}V_{DC}$. The collapse of the traces onto a single curve demonstrates that spin pumping is governed by the induced current I rather than the explicit applied bias voltage V_{DC}.

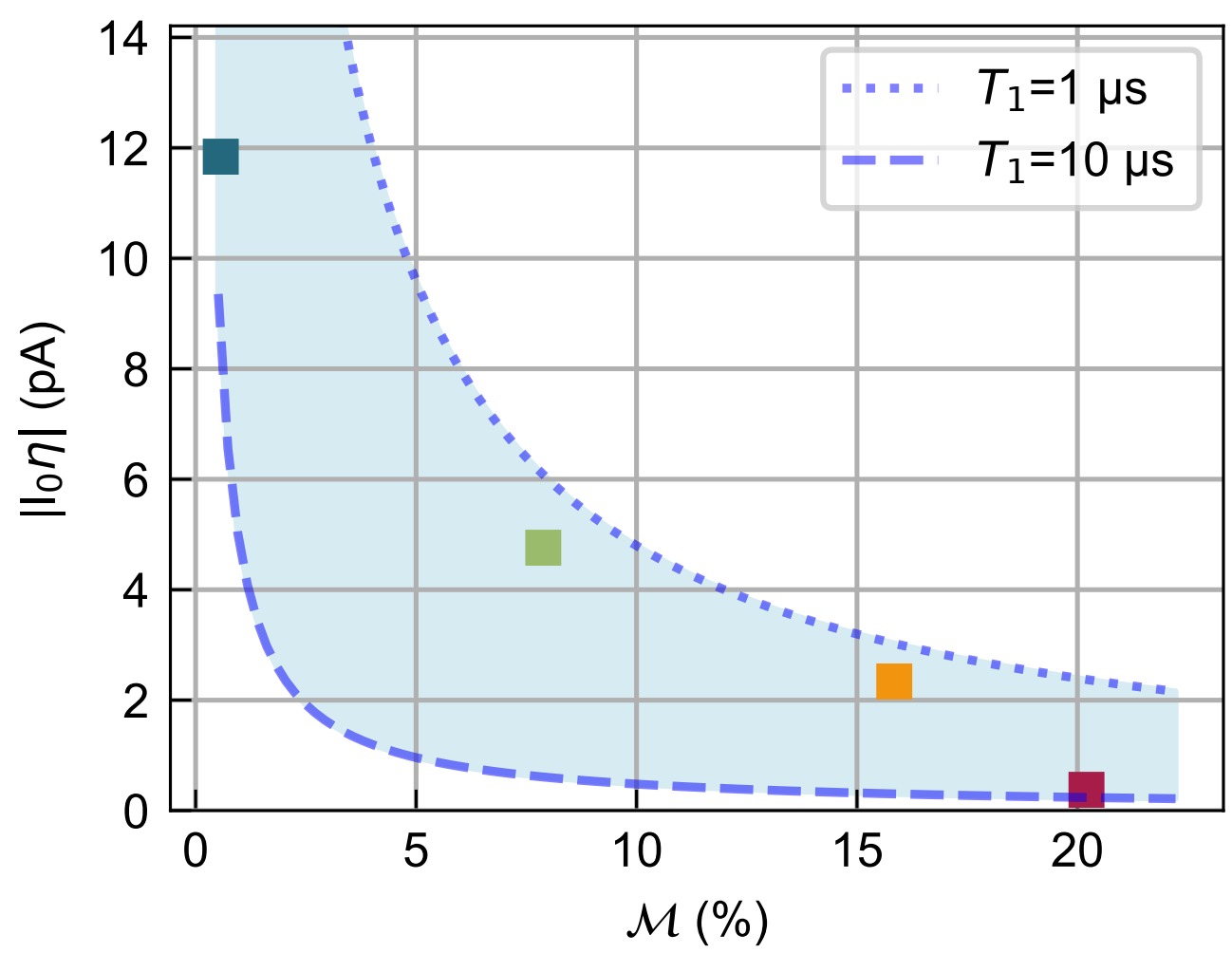

Figure S8: **Lifetime Estimation.** $|I_0\eta|$ as a function of $\mathcal{M}$. Squares are experimental values obtained from the measurements presented in Fig. 4 showing an inverse dependence. The dotted (dashed) line represents the behavior expected from the analytical approximation for $T_1 = 1\ \mu$s ($T_1 = 10\ \mu$s).

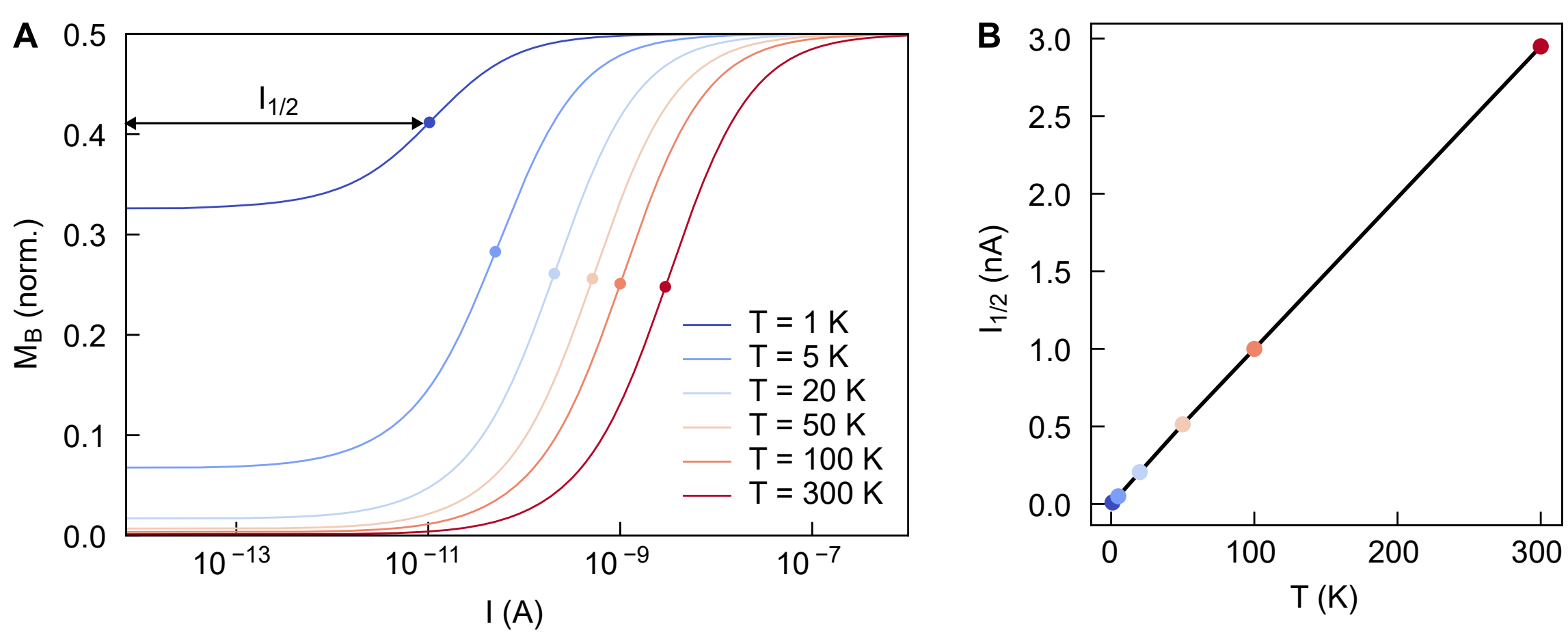

Figure S9: **Spin pumping for different temperatures.** (**A**) Magnetization of the remote spin M_B as a function of tunneling current I for different temperatures. For higher temperatures more current is required to magnetize the remote spin. This is quantified by $I_{1/2}$ which is defined as: $M_\mathrm{B}(I_{1/2}) = M_\mathrm{B}(0) + \frac{1}{2}[\eta - M_\mathrm{B}(0)]$ (dots along the lines). (**B**) $I_{1/2}$ over T extracted from the traces in (A) showing a linear behavior. [Simulation parameters: $g = 2.0$, $B_\mathrm{tip} = 36$ mT, $B = 500$ mT, $J_\parallel = -200$ MHz, $J_\perp = 300$ MHz, $\eta = 0.5$, $G_{st} = 0.2$ nS, $G_{ss} = 5$ nS].

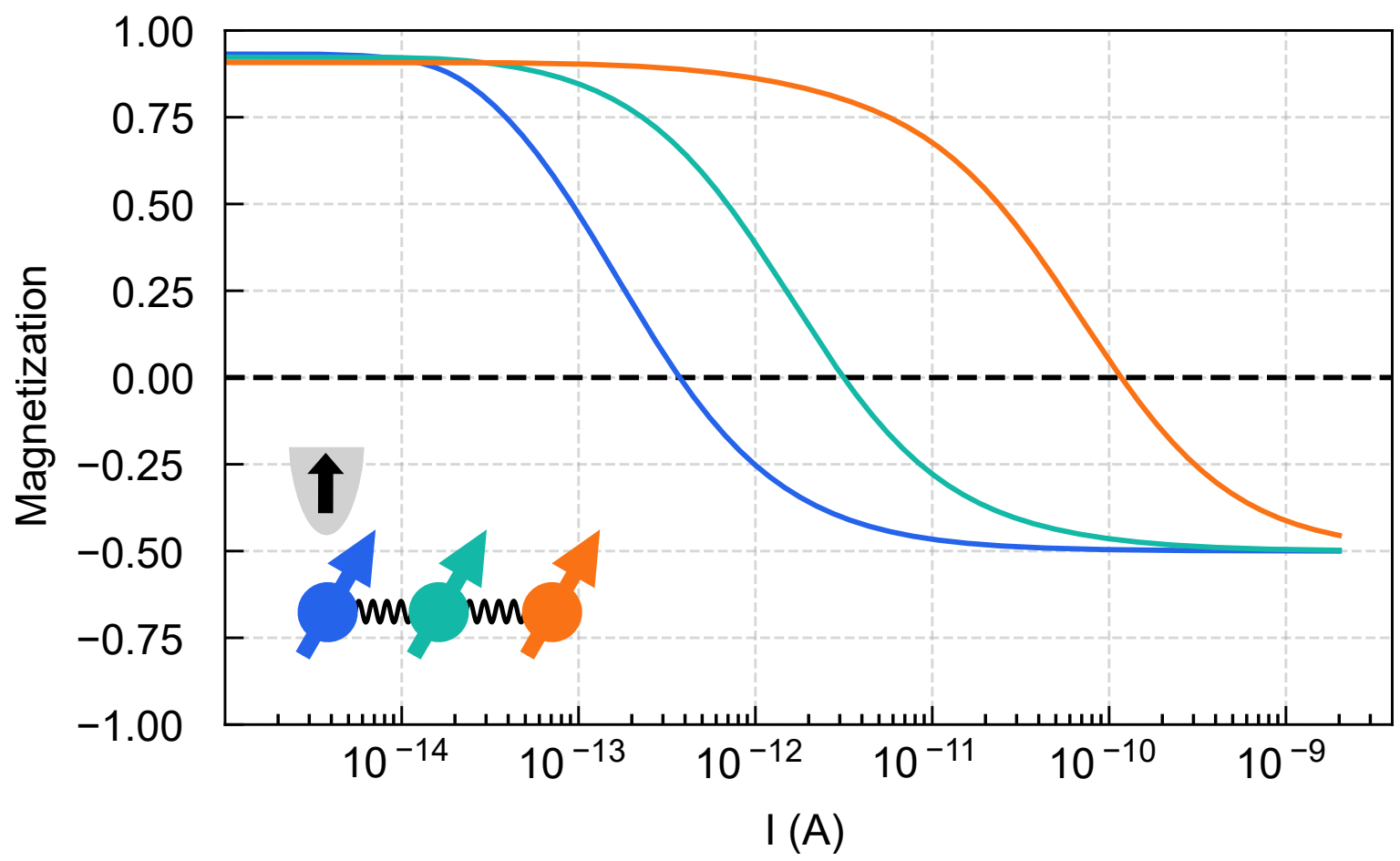

Figure S10: **Spin pumping simulation of a three spin chain.** Magnetization of the three spins plotted over the applied spin-polarized tunneling current I (logarithmic scale). The geometry of the spin system is indicated by the sketch inserted at the lower left. The results show that for a sufficiently high tunneling current also a next-nearest-neighbour spin can be polarized. [Simulation parameters: $g_{\mathrm{A}} = 2.0, g_{\mathrm{B}} = 1.9, g_{\mathrm{C}} = 1.8, B = 500$ mT, $T = 200$ mK, $J_{\|} = -200$ MHz, $J_{\perp} = 300$ MHz, $\eta = -0.5, G_{st} = 0.2$ nS, $G_{ss} = 5$ nS].

Molecule Pair	blue	green	yellow	red
$\lvert J_\perp \rvert$ (MHz)	220 ± 3	329 ± 5	521 ± 5	621 ± 3
$J_\parallel$ (MHz)	−135 ± 2	457 ± 5	−220 ± 1	−403 ± 1
δ (MHz)	2049 ± 2	769 ± 2	790 ± 3	791 ± 2

Table S1: **Coupling Parameters.** Summary of the exchange interaction parameters $|J_\perp|, J_\parallel$ and the detuning δ determined from the measurements shown in Fig. 4B,C.

REFERENCES

1. D. P. DiVincenzo, The physical implementation of quantum computation. *Fortschr. Phys.* **48**, 771–783 (2000).

2. J. M. Elzerman, R. Hanson, L. H. Willems van Beveren, B. Witkamp, L. M. K. Vandersypen, L. P. Kouwenhoven, Single-shot read-out of an individual electron spin in a quantum dot. *Nature* **430**, 431–435 (2004).

3. T. Chakraborty, J. Zhang, D. Suter, Polarizing the electronic and nuclear spin of the NV-center in diamond in arbitrary magnetic fields: Analysis of the optical pumping process. *New J. Phys.* **19**, 073030 (2017).

4. J. I. Cirac, P. Zoller, Quantum computations with cold trapped ions. *Phys. Rev. Lett.* **74**, 4091–4094 (1995).

5. M. S. Fataftah, D. E. Freedman, Progress towards creating optically addressable molecular qubits. *Chem. Commun.* **54**, 13773–13781 (2018).

6. E. Vasilenko, V. Unni Chorakkunnath, J. Resch, N. Jobbitt, D. Serrano, P. Goldner, S. K. Kuppusamy, M. Ruben, D. Hunger, Optically detected nuclear magnetic resonance of coherent spins in a molecular complex. *Nat. Mater.* **25**, 1154–1159 (2026).

7. S. Roggors, T. Unden, A. Aubele, P. Mentzel, G. Bayer, A. Salhov, J. Scharpf, M. B. Plenio, A. Retzker, F. Jelezko, T. R. Eichhorn, T. A. Schaub, M. Pfender, P. Neumann, I. Schwartz, A single-molecule spin-photon interface. arXiv:2605.10077 [quant-ph] (2026).

8. M. Atzori, R. Sessoli, The second quantum revolution: Role and challenges of molecular chemistry. *J. Am. Chem. Soc.* **141**, 11339–11352 (2019).

9. A. Gaita-Ariño, F. Luis, S. Hill, E. Coronado, Molecular spins for quantum computation. *Nat. Chem.* **11**, 301–309 (2019).

10. E. Moreno-Pineda, C. Godfrin, F. Balestro, W. Wernsdorfer, M. Ruben, Molecular spin qudits for quantum algorithms. *Chem. Soc. Rev.* **47**, 501–513 (2018).

11. A. Chiesa, P. Santini, E. Garlatti, F. Luis, S. Carretta, Molecular nanomagnets: A viable path toward quantum information processing? *Rep. Prog. Phys.* **87**, 034501 (2024).

12. S. Baumann, W. Paul, T. Choi, C. P. Lutz, A. Ardavan, A. J. Heinrich, Electron paramagnetic resonance of individual atoms on a surface. *Science* **350**, 417–420 (2015).

13. K. Yang, W. Paul, S.-H. Phark, P. Willke, Y. Bae, T. Choi, T. Esat, A. Ardavan, A. J. Heinrich, C. P. Lutz, Coherent spin manipulation of individual atoms on a surface. *Science* **366**, 509–512 (2019).

14. Y. Wang, Y. Chen, H. T. Bui, C. Wolf, M. Haze, C. Mier, J. Kim, D.-J. Choi, C. P. Lutz, Y. Bae, S.-H. Phark, A. J. Heinrich, An atomic-scale multi-qubit platform. *Science* **382**, 87–92 (2023).

15. X. Zhang, C. Wolf, Y. Wang, H. Aubin, T. Bilgeri, P. Willke, A. J. Heinrich, T. Choi, Electron spin resonance of single iron phthalocyanine molecules and role of their non-localized spins in magnetic interactions. *Nat. Chem.* **14**, 59–65 (2022).

16. P. Willke, T. Bilgeri, X. Zhang, Y. Wang, C. Wolf, H. Aubin, A. Heinrich, T. Choi, Coherent spin control of single molecules on a surface. *ACS Nano* **15**, 17959–17965 (2021).

17. S. Kovarik, R. Schlitz, A. Vishwakarma, D. Ruckert, P. Gambardella, S. Stepanow, Spin torque-driven electron paramagnetic resonance of a single spin in a pentacene molecule. *Science* **384**, 1368–1373 (2024).

18. K. H. Au-Yeung, W. Huang, J. Matusche, P. Greule, J. Arnold, L. Hardeweg, M. Stark, L. Renz, A. Safeer, D. Jansen, T. Michely, J. Fischer, W. Wernsdorfer, C. Sürgers, H.-P. Komsa, J. Schwenk, W. Jolie, P. Willke, Atomic-scale quantum control of single spin defects in a two-dimensional semiconductor. arXiv:2602.22301 [cond-mat.mes-hall] (2026).

19. K. Yang, P. Willke, Y. Bae, A. Ferrón, J. L. Lado, A. Ardavan, J. Fernández-Rossier, A. J. Heinrich, C. P. Lutz, Electrically controlled nuclear polarization of individual atoms. *Nat. Nanotechnol.* **13**, 1120–1125 (2018).

20. L. M. Veldman, E. W. Stolte, M. P. Canavan, R. Broekhoven, P. Willke, L. Farinacci, S. Otte, Coherent spin dynamics between electron and nucleus within a single atom. *Nat. Commun.* **15**, 7951 (2024).

21. E. W. Stolte, J. Lee, H. G. Vennema, R. Broekhoven, E. Teng, A. J. Katan, L. M. Veldman, P. Willke, S. Otte, Single-shot readout of the nuclear spin of an on-surface atom. *Nat. Commun.* **16**, 7785 (2025).

22. W. Paul, K. Yang, S. Baumann, N. Romming, T. Choi, C. P. Lutz, A. J. Heinrich, Control of the millisecond spin lifetime of an electrically probed atom. *Nat. Phys.* **13**, 403–407 (2017).

23. W. Huang, M. Stark, P. Greule, K. H. Au-Yeung, D. Sostina, J. Reina Gálvez, C. Sürgers, W. Wernsdorfer, C. Wolf, P. Willke, Quantum spin-engineering in on-surface molecular ferrimagnets. *Nat. Commun.* **16**, 5208 (2025).

24. P. Greule, W. Huang, M. Stark, K. H. Au-Yeung, J. Schwenk, J. Reina-Gálvez, C. Sürgers, W. Wernsdorfer, C. Wolf, P. Willke, Exchange-mediated spin-electric control of single molecules on surfaces. *Nat. Phys.* **22**, 1394–1400 (2026).

25. K. Yang, Y. Bae, W. Paul, F. D. Natterer, P. Willke, J. L. Lado, A. Ferrón, T. Choi, J. Fernández-Rossier, A. J. Heinrich, C. P. Lutz, Engineering the eigenstates of coupled spin-1/2 atoms on a surface. *Phys. Rev. Lett.* **119**, 227206 (2017).

26. L. M. Veldman, L. Farinacci, R. Rejali, R. Broekhoven, J. Gobeil, D. Coffey, M. Ternes, A. F. Otte, Free coherent evolution of a coupled atomic spin system initialized by electron scattering. *Science* **372**, 964–968 (2021).

27. T. Choi, W. Paul, S. Rolf-Pissarczyk, A. J. Macdonald, F. D. Natterer, K. Yang, P. Willke, C. P. Lutz, A. J. Heinrich, Atomic-scale sensing of the magnetic dipolar field from single atoms. *Nat. Nanotechnol.* **12**, 420–424 (2017).

28. Y. del Castillo, J. Fernández-Rossier, Theory of atomic-scale direct thermometry using electron spin resonance via scanning tunneling microscopy. *Nano Lett.* **25**, 2159–2165 (2025).

29. P. Greule, J. Arnold, L. Renz, L. Hardeweg, J. Matusche, Spinfinity: A Python package for simulating quantum spin systems in tunnel junction experiments, GitHub (2026); https://github.com/WillkeLab/Spinfinity.

30. S. Loth, K. von Bergmann, M. Ternes, A. F. Otte, C. P. Lutz, A. J. Heinrich, Controlling the state of quantum spins with electric currents. *Nat. Phys.* **6**, 340–344 (2010).

31. M. Ternes, Spin excitations and correlations in scanning tunneling spectroscopy. *New J. Phys.* **17**, 063016 (2015).

32. S. Loth, C. P. Lutz, A. J. Heinrich, Spin-polarized spin excitation spectroscopy. *New J. Phys.* **12**, 125021 (2010).

33. F. Delgado, J. J. Palacios, J. Fernández-Rossier, Spin-transfer torque on a single magnetic adatom. *Phys. Rev. Lett.* **104**, 026601 (2010).

34. C. Wolf, A. J. Heinrich, S.-H. Phark, On-surface atomic scale qubit platform. *ACS Nano* **18**, 28469–28479 (2024).

35. S. R. McMillan, N. J. Harmon, M. E. Flatté, Image of dynamic local exchange interactions in the dc magnetoresistance of spin-polarized current through a dopant. *Phys. Rev. Lett.* **125**, 257203 (2020).

36. J. Yu, C. Urdaniz, Y. Namgoong, C. Wolf, On the magnetic bistability of small iron clusters used in scanning tunneling microscopy tip preparation. *New J. Phys.* **25**, 113035 (2023).

37. D. Wu, X. Geng, X. Wu, D. Xuan, S. Li, B. Esmaeilzadeh, Y. Hu, C. Wolf, X. Zhang, Atomic-scale mapping of spin redistribution in chemically engineered metal-organic complexes. *J. Phys. Chem. Lett.* **17**, 4408–4417 (2026).

38. Y. Bae, M. Ternes, K. Yang, A. J. Heinrich, C. Wolf, C. P. Lutz, Direct observation of fully spin-polarized tunnel current between quantum spins using a single molecule sensor. *ACS Nano* **19**, 1361–1370 (2025).

39. T. Machida, High-resolution spin-polarized scanning tunneling spectroscopy using a functionalized superconducting tip. *Microscopy* **75**, 229–239 (2026).

40. D. Wang, J. Wiebe, R. Zhong, G. Gu, R. Wiesendanger, Spin-polarized Yu-Shiba-Rusinov states in an iron-based superconductor. *Phys. Rev. Lett.* **126**, 076802 (2021).

41. A. Gruber, A. Dräbenstedt, C. Tietz, L. Fleury, J. Wrachtrup, C. von Borczyskowski, Scanning confocal optical microscopy and magnetic resonance on single defect centers. *Science* **276**, 2012–2014 (1997).

42. P. Greule, W. Huang, K. H. Au-Yeung, M. Stark, J. Schwenk, C. Suergers, W. Wernsdorfer, P. Willke, Electrical spin pumping in exchange-coupled molecules, Zenodo (2026); https://doi.org/10.5281/zenodo.21644138.

43. R. Wiesendanger, H. Güntherodt, G. Güntherodt, R. J. Gambino, R. Ruf, Observation of vacuum tunneling of spin-polarized electrons with the scanning tunneling microscope. *Phys. Rev. Lett.* **65**, 247–250 (1990).
